# Centripetal integration of past events in hippocampal astrocytes regulated by locus coeruleus

**DOI:** 10.1038/s41593-024-01612-8

**Published:** 2024-04-03

**Authors:** Peter Rupprecht, Sian N. Duss, Denise Becker, Christopher M. Lewis, Johannes Bohacek, Fritjof Helmchen

**Affiliations:** 1 Laboratory of Neural Circuit Dynamics, Brain Research Institute, University of Zurich, Zürich, Switzerland; 2 Neuroscience Center Zurich, University of Zurich and ETH Zurich, Zürich, Switzerland; 3 Laboratory of Molecular and Behavioral Neuroscience, Institute for Neuroscience, Department of Health Sciences and Technology, ETH Zurich, Zürich, Switzerland; 4 University Research Priority Program (URPP), Adaptive Brain Circuits in Development and Learning, University of Zurich, Zürich, Switzerland

**Keywords:** Astrocyte, Neural circuits, Reward

## Abstract

An essential feature of neurons is their ability to centrally integrate information from their dendrites. The activity of astrocytes, in contrast, has been described as mostly uncoordinated across cellular compartments without clear central integration. Here we report conditional integration of calcium signals in astrocytic distal processes at their soma. In the hippocampus of adult mice of both sexes, we found that global astrocytic activity, as recorded with population calcium imaging, reflected past neuronal and behavioral events on a timescale of seconds. Salient past events, indicated by pupil dilations, facilitated the propagation of calcium signals from distal processes to the soma. Centripetal propagation to the soma was reproduced by optogenetic activation of the locus coeruleus, a key regulator of arousal, and reduced by pharmacological inhibition of α1-adrenergic receptors. Together, our results suggest that astrocytes are computational units of the brain that slowly and conditionally integrate calcium signals upon behaviorally relevant events.

## Abstract (web summary)

How astrocytes can integrate information is incompletely understood. Here the authors show that locus coeruleus-controlled calcium signals in hippocampal astrocytes propagating from their processes to their soma are involved in the information integration upon salient events.

## Main

Astrocytes have long been associated with supportive rather than computational functions in the brain. More recently, studies started to assign computational roles to astrocytes, as for neurons^1,2^. For example, astrocytic calcium signals were proposed to represent sensory-related or internally generated information^3–6^. In contrast to neurons, however, calcium signals are thought to be mostly uncoordinated across the compartments of a single astrocyte^7–9^. It is therefore not clear whether and how astrocytes integrate signals that are sensed by their distributed compartments. The study of signal integration in astrocytes is particularly challenging because they express a large set of receptors to sense the direct and indirect effects of neuronal activity and neuromodulation^7,10^, which both interact with behavior. It is therefore essential to study astrocytic activity and signal integration in astrocytes together with such correlated factors, for example, neuromodulatory signals^5,11,12^, pupil diameter^13^, locomotion^12,14–16^ and neuronal activity^4,8,9^.

Here we perform a systematic exploration of astrocytic activity in hippocampal CA1 using two-photon calcium imaging in behaving mice. We find that global population activity can be described as a temporal integration of past events such as unexpected air puffs or self-generated movement. On the single-astrocyte level, this integration manifests as calcium signals that spatially propagate from distal processes to the soma. Spontaneous-behavior analysis as well as perturbations based on optogenetics and pharmacology suggest that this centripetal propagation is gated by noradrenaline release from the locus coeruleus (LC). Together, our observations reveal a principle of spatiotemporal integration of salient past events within astrocytes in the awake, behaving animal.

## Results

### Synchronized global astrocytic activity in awake mice

**Fig. 1:**
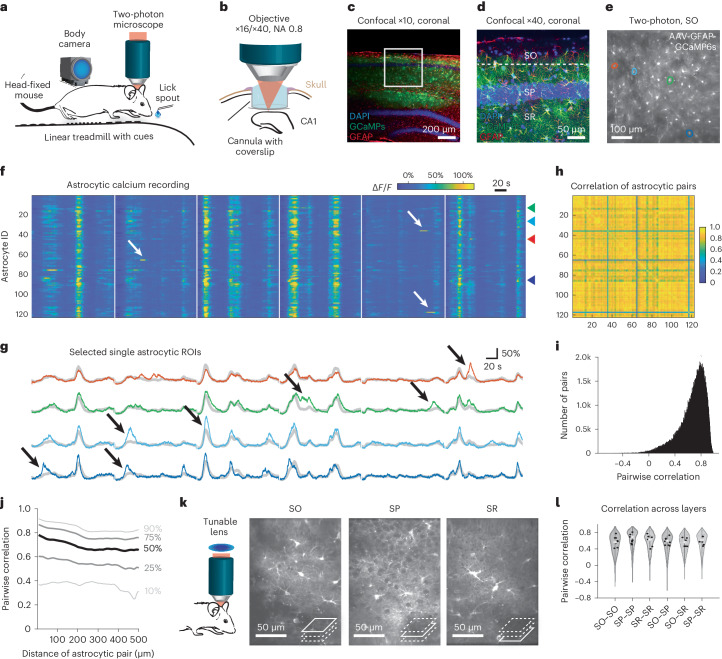
Hippocampal astrocytes in CA1 exhibit global and local events during behavior. **a**, Schematic representation of in vivo recording setup. **b**, Hippocampal two-photon imaging through an implanted cannula. **c**,**d**, Histology of virus-induced GCaMP6s expression in hippocampal astrocytes (green) together with GFAP-antibody staining (red) and nuclear stain (blue). Overview (**c**) and zoom-in (**d**). See also Extended Data Fig. 1 and Supplementary Fig. 2. **e**, Average fluorescence two-photon image of astrocytes in the SO expressing GCaMP6s. **f**, Temporal calcium dynamics of active astrocyte ROIs from the FOV shown in **e**. White arrows indicate isolated local calcium events. Recording segments (140 s) are indicated through vertical white spacers. **g**, Example of four astrocytic ROIs (highlighted with matching colors in **e** and in **f** with arrowheads), indicating local modulation of global events for astrocytic ROIs (black arrows). Global mean across the FOV is overlaid as gray traces. **h**, Activity correlation between the astrocytic pairs from **f**, same ordering of ROIs. **i**, Distribution of activity correlations between astrocytic active region pairs across the entire population (204,686 astrocyte ROI pairs from 41 experimental sessions and six animals; 0.72 ± 0.20, median ± s.d.). **j**, Distance dependence of pairwise correlations, with the median (50%) and other percentile lines of the distribution shown. **k**, Using a tunable lens to quasi-simultaneously image multiple CA1 layers. **l**, Pairwise correlation across simultaneously imaged astrocyte pairs associated with specific CA1 layers (distributions in the violin plots), and medians across astrocyte pairs for each session (black dots). No significant differences (*P* > 0.2 for all comparisons) across conditions for session-based testing (*n* = 8 sessions from two animals). Source data

**Extended Data Fig. 1:**
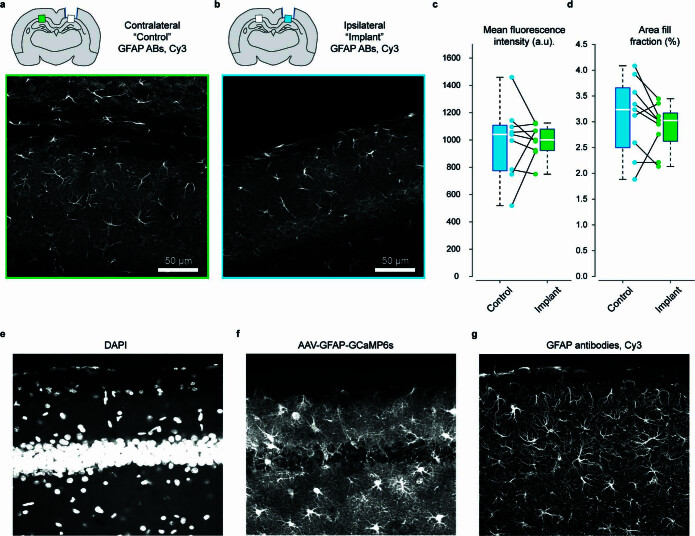
Histology of GFAP expression in astrocytes. **a**,**b**, Examples of GFAP expression in CA1 stratum oriens from the same animal contralateral (**a**) and ipsilateral (**b**) to the injection and cannula implantation site. **c**, Quantification of mean fluorescence intensity in regions of interest drawn across the imaging site (stratum oriens), compared within the same slice. Nine slices from 3 animals. No statistically significant difference was observed (Wilcoxon signed-rank test). **d**, Quantification of area covered by astrocytes (thresholding; see Methods) within selected regions of interest. The same threshold was applied to paired images from the same slice. **e**–**g**, Histology channels of Fig. 1d, shown separately in grayscale. **e**, Nuclear stain DAPI. **f**, Virus-induced GCaMP6s expression in hippocampal astrocytes. **g**, GFAP-antibody staining of astrocytes. Maximum intensity projection across 12 µm. While antibodies tended to more heavily stain the distal processes of astrocytes, all astrocytes labeled with the GFAP antibody also exhibited expression with AAV-induced GCaMP6s. Source data

To record astrocytic activity in the hippocampus across a wide range of behaviors, we virally induced expression of GCaMP6s (*n* = 6 mice; AAV9-GFAP-GCaMP6s; Extended Data Fig. 1) and performed two-photon calcium imaging in head-fixed mice. Mice were free to run on a treadmill and received water rewards at a defined location, resulting in variable behavior including periods of active running and quietness (Fig. 1a). First, we analyzed calcium dynamics in the astrocytic population in stratum oriens (SO) of CA1 (Fig. 1b–e and Supplementary Video 1). Across active regions of interest (ROIs; Supplementary Fig. 1), astrocytic calcium signals were highly correlated (Fig. 1f–i). Therefore, the global astrocytic activity, defined as average fluorescence trace across the field of view (FOV), explained a large fraction of the variance of single astrocyte activity (Fig. 1h,i; correlation: 0.72 ± 0.20, median ± s.d. across 204,686 pairs of astrocytes from 41 sessions and six mice). Pairwise correlations decayed only slightly with distance between astrocytes (Fig. 1j), indicating a global rather than local synchronization within hippocampal CA1. In addition, we observed local events in single astrocytes independent of global activity (Fig. 1f (white arrows)) and modulations during global activity specific to single astrocytes (Fig. 1g (black arrows) and Supplementary Video 1). However, the overall activity during behavior was dominated by a global mode across astrocytes.

To study astrocytic activity across different depths of hippocampal CA1, we performed triple-layer calcium imaging using fast z-scanning with a tunable lens (Methods). With this approach, we could image quasi-simultaneously from astrocytes in SO, stratum pyramidale (SP) and stratum radiatum (SR) (Fig. 1k and Supplementary Video 2). Astrocytic activity was highly correlated across layers (Fig. 1l) with astrocytic pairs displaying similar correlation across and within layers (*P* > 0.2 for all comparisons, Wilcoxon’s rank-sum test, *n* = 8 sessions from two animals). Due to the sparseness of astrocytic somata in SP^17^ and the better imaging access to SO, we focused all remaining experiments on SO.

### Pupil dynamics, body movements and neuronal activity explain astrocytic activity

**Fig. 2:**
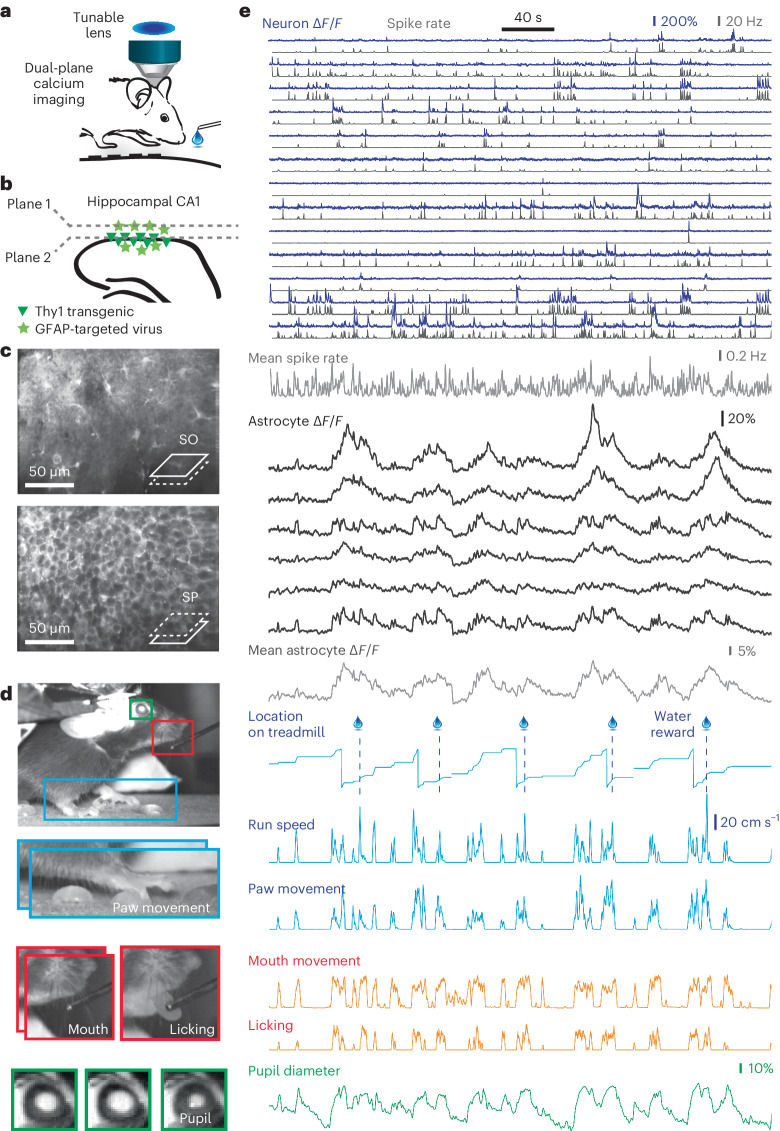
Simultaneous monitoring of astrocytic and neuronal population activity, pupil diameter and behavior. **a**, Dual-plane calcium imaging using a tunable lens. **b**, Simultaneously imaging of spatially separated astrocytes (SO layer, virally induced GCaMP6s; star symbols) and transgenically expressed neurons (SP layer, Thy1-GCaMP6f; triangle symbols). **c**, Mean fluorescence image of simultaneously imaged astrocytes in SO and neurons in SP. **d**, Example behavioral camera image. Blue, paw movement extracted from subsequent video frames. Red, mouth movement extracted from subsequent video frames, licking extracted by tongue detection. Green, pupil diameter visible due to laser light passing from the brain through the eye. **e**, Example of simultaneous recordings, from top to bottom, subset of neuronal Δ*F*/*F* traces (blue, 13 from a total of 107 neurons) extracted from FOV in **c**, together with deconvolved spike rates (black) and mean spike rate across all 107 neurons (bottom). Extracted astrocytic Δ*F*/*F* traces (6 of total 34 active astrocytic ROIs), together with the mean astrocytic trace across the FOV in **c**. Blue, tracking of the position along the treadmill, together with timepoints of water rewards; run speed is determined from a rotary encoder, and paw movement is extracted from video analysis (blue panel in **d**). Red, mouth movement and licking are extracted from video analysis (red panels in **d**). Green, pupil diameter, relative change with respect to median, extracted from video analysis (green panel in **d**). Source data

To relate astrocytic activity to behavior and neuronal activity, we simultaneously imaged astrocytes in SO (virally induced GCaMP6s as before) and neurons in the SP, 60–90 µm below the SO-imaging plane in transgenic Thy1-GCaMP6f mice (Fig. 2a–c; *n* = 4 mice, 22 imaging sessions). GCaMP signals of astrocytes and neurons were clearly distinct due to their different location (SO versus SP) and dynamics (slow versus fast transients). Within SO, we additionally unmixed neuronal and glial signals (Supplementary Fig. 2; Methods). To quantify average neuronal population activity, we denoised Δ*F*/*F* traces for neuronal ROIs using a supervised deconvolution algorithm^18^ and obtained an overall spike rate estimate by averaging denoised spike rates. For behavioral analysis, we tracked run speed, location on the treadmill, pupil dynamics and body movements (Fig. 2d). From video recordings, we estimated the movement of mouth and paws using across-frame correlation and quantified licking by detecting the tongue (Methods). In addition, we segmented the eye pupil and tracked pupil diameter as a proxy for neuromodulatory tone and arousal^19^. These multiple perspectives (Fig. 2e and Supplementary Video 3) enable a comprehensive analysis of astrocytic activity in the context of diverse, possibly related processes and events.

**Fig. 3:**
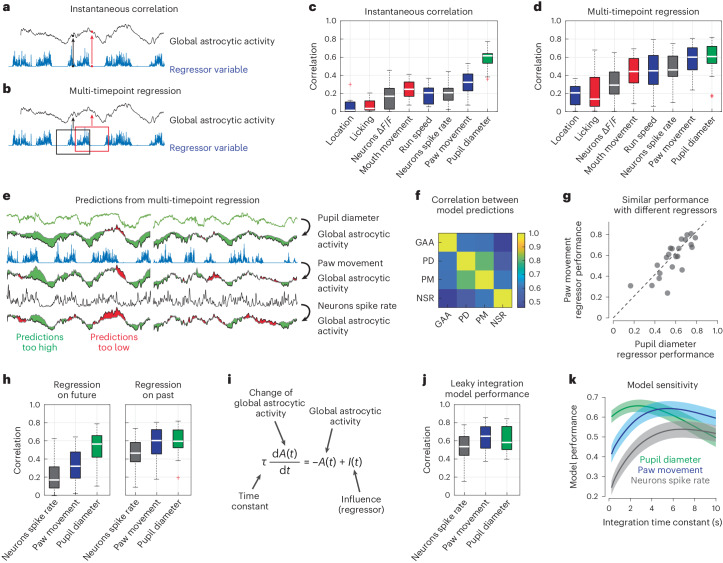
Global astrocytic activity can be well explained by past behavior, mean neuronal spike rate or pupil diameter. **a**, Schematic illustration of instantaneous correlation. A single timepoint is used to predict a simultaneous astrocytic timepoint. **b**, Schematic illustration of multi-timepoint regression. A range of data points in a window is used to predict a single timepoint of astrocytic activity. **c**,**d**, Performance of various regressors when using instantaneous correlation (**c**) or multi-timepoint analysis (**d**) to predict astrocytic activity (*n* = 22 imaging sessions from four animals). Box plot properties are defined as described in Methods. **e**, Example cross-validated predictions from the three best regressors (pupil diameter, paw movement and mean neuronal spike rate). Green areas indicate when predicted activity is too high, and red areas indicate when it is too low. **f**, Average correlation between global astrocytic activity and the activity predicted by the three regressors (GAA, global astrocytic activity; PD, pupil diameter; PM, paw movement; NSR, neurons spike rate). **g**, Example of performance for two different regressors (pupil diameter and paw movement) across sessions (correlation *r* = 0.79). See also Supplementary Fig. 5. **h**, Multi-timepoint regression, but using only either future or past timepoints (*n* = 22 sessions). **i**, Leaky integration differential equation to model global astrocytic activity *A*(*t*) as a function of regressor input *I*(*t*), dependent on the integration time constant *τ*. **j**, Performance using the leaky integration differential equation (*n* = 22 sessions from four animals). **k**, Model sensitivity with respect to *τ* (mean ± bootstrapped 90% confidence interval of the mean, determined from 22 imaging sessions). For box plots, the median is indicated by the central line; 25th and 75th percentiles are indicated by the box and maximum/minimum values excluding outliers are indicated by the whiskers. Source data

Due to the slow changes in global astrocytic activity, our recordings only sparsely sampled the state space of astrocytic activity. Given this limitation, we first aimed to explain not single-cell activity but only the global mode of astrocytic activity, using either run speed, body movements, pupil diameter or mean neuronal spike rate as explanatory variables. Estimating the shared information between global astrocytic activity and these factors using instantaneous correlation (Fig. 3a), we found the highest correlation with pupil diameter, whereas run speed, body movements and neuronal activity were less correlated (Fig. 3c). Next, to account for possible delayed effects that cannot be captured by correlation or other instantaneous measures, we used past and future timepoints of neuronal and behavioral variables to explain the current value of global astrocytic activity using multi-timepoint, dilated linear regression (Fig. 3b; Methods). With this analysis, the time courses predicted by paw movement or neuronal spike rate explained global astrocytic activity almost equally well as pupil diameter (Fig. 3d). Notably, the mean neuronal spike rate was a better predictor than the average Δ*F*/*F* trace before deconvolution, due to the denoising property of deconvolution^18^. Paw movement was a better predictor than mouth movement and much better than licking alone (Fig. 2d–e). These findings are consistent with observations from behavioral monitoring that mouth-only movements did not reliably evoke astrocytic responses (Supplementary Video 4). Analysis of additional experiments recorded from astrocytes without neuronal imaging confirmed these results (Supplementary Fig. 3).

Based on these findings, we focus in following analyses on pupil diameter, paw movement and mean neuronal spike rate as the best predictors of global astrocytic activity. Overall, predictions were not significantly improved when using multiple or higher-dimensional regressors for dilated regression (Supplementary Fig. 4), suggesting mostly redundant regressors. Consistent with this notion, predictions based on the different regressors were highly correlated (Fig. 3e). For example, predictions based on paw movement and pupil showed an even higher correlation among each other than with the global astrocytic activity itself (Fig. 3f; 0.76 ± 0.14 versus 0.59 ± 0.15, mean ± s.d.; *P* = 0.00026, Wilcoxon signed-rank test). In addition, two regressors typically performed similarly for the same imaging session but covaried across sessions (Fig. 3g and Supplementary Fig. 5). Furthermore, regressors were able to mutually explain each other by multi-timepoint dilated regression (Supplementary Fig. 6). Together, these analyses highlight that seemingly unrelated behavioral and neuronal variables can explain global astrocytic activity equally well when noninstantaneous dependencies are considered.

### Global astrocytic activity as a leaky integration of past events

Next, to better understand the performance of multi-timepoint models, we repeated the regression analysis but used only past or future regressor timepoints to predict global astrocytic activity. We found that past but not future timepoints of paw movement or neuronal spike rate could be used to predict astrocytic calcium transients (Fig. 3h). For pupil diameter, we found a less striking difference between predictors based on past versus future timepoints (Fig. 3h). Consistent with this, the pupil-based multi-timepoint model did not exhibit a significant performance increase with respect to instantaneous correlation (Fig. 3c,d; *P*_boot_ = 0.53, hierarchical bootstrapping test with 22 sessions from four mice). Following up on these results, we attempted to model global astrocytic activity as a temporal integration of mean neuronal spike rate, paw movement or pupil diameter changes. Specifically, we fitted a linear differential equation that simulates global astrocytic activity as a leaky integration of a single variable, with a single free parameter, the time constant *τ* (Fig. 3i). This simple model showed a trend toward explaining an even higher amount of variance than the regression for the paw movement and neuronal activity regressors (Fig. 3j versus Fig. 3d; *P*_boot_ = 0.061 and 0.041 for neuronal spike rate and paw movement, *P*_boot_ = 0.75 for pupil). The integration time constants that were obtained as fit parameters were relatively short for pupil diameter (*τ* = 2.8 ± 0.5 s; mean ± 90% bootstrapped confidence intervals across sessions) but substantially longer for paw movement (5.6 ± 0.5 s) and neuronal spike rate (6.8 ± 0.5 s), with a relatively weak sensitivity of the model for changes of the specific time constant (Fig. 3k). We conclude that global astrocytic activity can be well described as a nearly instantaneous readout of pupil diameter but, alternatively, also as a leaky integration of past neuronal population spike rate or body movement.

### Past events rather than spatial landmarks explain astrocytic activity

**Extended Data Fig. 2:**
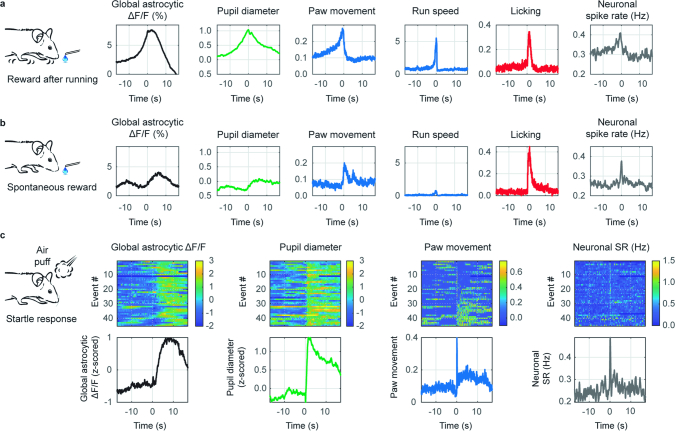
Pupil diameter but not body movement is reliably coupled to global astrocytic events. **a**, Event-triggered average traces for rewards that are obtained at a reward location after running, aligned to reward consumption (average across n = 260 events). All variables with arbitrary scaling (a.u.) unless otherwise indicated. **b**, Event-triggered average traces for spontaneous rewards, aligned to reward consumption (n = 168). All variables with arbitrary scaling (a.u.) unless otherwise indicated. **c**, Event-triggered traces for air-puff-induced startle responses (top: heatmap of individual traces for 45 events; bottom: average across events). Single traces for ΔF/F and pupil diameter are z-scored. Traces in heatmaps are sorted by increasing paw movement during the first 3 s after the air puff. Source data

Hippocampal astrocytes have been proposed to show activation primarily during phases of reward expectation during spatial navigation^6^. We examined this hypothesis and compared it to our seemingly contradicting finding that astrocytic activity is explained by past events, by inspecting a set of externally generated and spontaneous behavioral events. We found that global astrocytic activity indeed ramped toward the location of an expected reward. However, this ramping occurred simultaneously with body movements, locomotion, neuronal activity and pupil diameter changes (Extended Data Fig. 2a). Increase of global astrocytic activity could also be seen upon randomly delivered rewards (Extended Data Fig. 2b). Furthermore, we observed that global astrocytic activity was better explained by past paw movement than by locomotion-associated run speed (Fig. 3c). We noticed that it was important to take into account past movement from delays of ≫2 s, which was not sufficiently considered in the previous study^6^ (Supplementary Fig. 7). Together, these observations suggest that astrocytic activity can be more parsimoniously explained as an integration of past events, rather than an encoding of future reward.

In support of this idea, a purely non-navigational behavior, when the mouse occasionally used its forepaws, consistently elicited an increase in global astrocytic activation despite lack of locomotion (Supplementary Fig. 8 and Supplementary Video 5). Moreover, we observed that behavioral events without significant body movement could elicit astrocytic activation: during some sessions, we applied an unpredictable air puff stimulus, as done previously^11,12,20^. For a subset of stimulus applications, the mouse remained immobile despite the stimulus, while the pupil diameter increased together with astrocytic activity (Extended Data Fig. 2c). These effects on astrocytes, which are unrelated to spatial landmarks, are in line with the idea established for other brain areas that increased global astrocytic activity is primarily triggered by arousal and mediated by noradrenergic neuromodulation^10^.

### Temporal sequence of events preceding global astrocytic activity

**Fig. 4:**
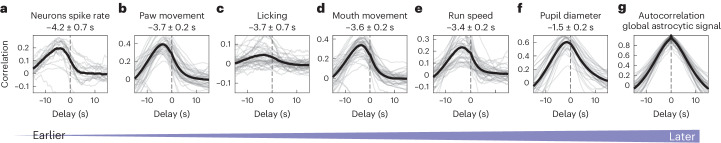
Temporal sequence of neurons spike rate, motor behaviors, pupil diameter and global astrocytic activity. **a**–**g**, Correlation functions were computed between the global astrocytic signal and the variable of interest: neuronal activity (**a**), paw movement (**b**), licking (**c**), mouth movement (**d**), run speed (**e**), pupil diameter (**f**) and astrocytic signal (**g**, autocorrelation). A peak of the correlation function with negative lag indicates that the inspected variable peaked on average earlier than global astrocytic activity. Gray traces are correlation functions extracted from single sessions, and black traces are averages across sessions. The delays indicated are median values ± s.e. across sessions (*n* = 41 sessions across six animals, except for pupil diameter with 33 sessions across six animals and neurons spike rate with 22 sessions across four animals). Source data

**Extended Data Fig. 3:**
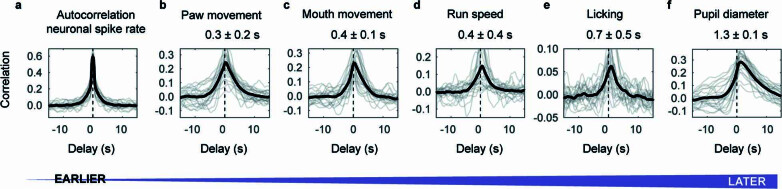
Temporal sequence of behavioral variables with respect to mean neuronal spike rate. **a-f**, Analogous to Fig. 4 but using deconvolved neuronal spike rate instead of global astrocytic activity as the reference signal, with autocorrelation of neuronal spike rate (**a**) and the crosscorrelation of neuronal spike rate with paw movement (**b**), mouth movement (**c**), run speed (**d**), licking (**e**), pupil diameter (**f**). A peak of the correlation function with positive lag indicates that the neuronal spike rate peaked on average earlier than the inspected variable. Gray traces are correlation functions extracted from single sessions, black traces are averages across sessions. The delays indicated are median values ± standard error across sessions (n = 22 sessions across 4 animals). Source data

Next, we quantified the temporal relationship of the observables functionally associated with astrocytic activity. We computed correlation functions, which enabled us to estimate the timing of any recorded observable relative to the global astrocytic calcium signal (Fig. 4a–g). Strikingly, we found a consistent temporal sequence as quantified by the peak of the correlation function. The deconvolved neuronal spike rate peaked first (−4.2 ± 0.7 s, before astrocytic calcium signal), followed by paw movements (−3.7 ± 0.2 s) and pupil diameter (−1.5 ± 0.2 s; see also Extended Data Fig. 3). Increases of the correlation functions for positive delays could suggest that global astrocytic activity might have downstream effects on the investigated variables. However, for the observed set of variables, we did not observe any such increases at positive lags (Fig. 4a–f). Overall, we found, on average, a consistent and stable sequence of events, from neuronal spike rate changes over various body movements and pupil diameter changes to astrocytic activation.

### Propagation of astrocytic activity from processes to the soma

**Fig. 5:**
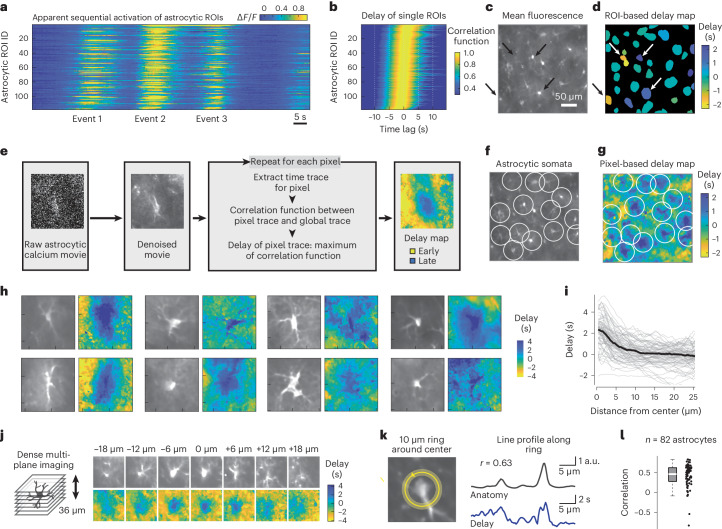
Propagation of astrocytic activity from distal to somatic compartments. **a**, Calcium signals of hippocampal astrocytes during behavior form an apparent sequence. Astrocytic ROIs are sorted by the delay of each signal with respect to the global mean. **b**, Correlation functions of astrocytic ROIs, same sorting as in **a**. The excerpt in **a** is only a fraction of the activity pattern used for the analysis. **c**, Excerpt from the mean fluorescence of the FOV from **a** and **b**. **d**, Delays extracted as maxima in **b**, mapped onto astrocytic ROIs. Rightward-pointing and leftward-pointing arrows highlight ROIs with negative (undefined anatomical structure) and positive delay (cell bodies), respectively. **e**. Processing pipeline for pixel-wise delay maps (Supplementary Fig. 9). **f**, Same map as in **c**, with white circles highlighting identified astrocytes. **g**, Pixel-based delay map corresponding (**f**). Somata (centers of circles) are activated with a positive delay and gliapil with a negative delay. **h**, Zoom-in to delay maps around identified astrocytes. The side length of each zoom-in is ~55 µm. See Extended Data Figs. 4 and 5 for more examples. **i**, Radial distribution of delay versus distance from astrocytic soma center (gray lines for individual astrocytes; 82 astrocytes from 11 sessions in four mice). **j**, Example of dense multiplane calcium imaging, FOV excerpt focused on a single astrocyte. The 3D delay map (computed from denoised data) exhibits the longest delay at the soma. The side length of each tile is ~55 µm. See Extended Data Fig. 6 for more examples. **k**, A 2.5-µm-thick ring with a diameter of 10 µm defines a circular line plot that covers both large (bright anatomy) as well as small (dark) processes. Delay and fluorescence (anatomy) along the circular line plot are visibly correlated. **l**, Distribution of Pearson correlation values as in **k** across 82 astrocytes (0.46 ± 0.29, median ± s.d.). For box plots, the median is indicated by the central line; 25th and 75th percentiles are indicated by the box and maximum/minimum values excluding outliers are indicated by the whiskers. Source data

To better understand astrocytic activity in single cells, we applied our analysis based on correlation functions also to the activity of single astrocytic ROIs (comprising either somatic or gliapil regions). Surprisingly, we observed that the delay of a given astrocytic ROI with respect to the global activity was variable across ROIs but consistent for a given ROI and could be a few seconds long (Fig. 5a,b). We investigated the spatial organization of such delays and found that ROIs with positive delays (later activation) tended to map onto regions comprising astrocytic somata, whereas ROIs with negative delays (earlier activation) mapped onto regions devoid of somata or large processes (Fig. 5c,d).

**Extended Data Fig. 4:**
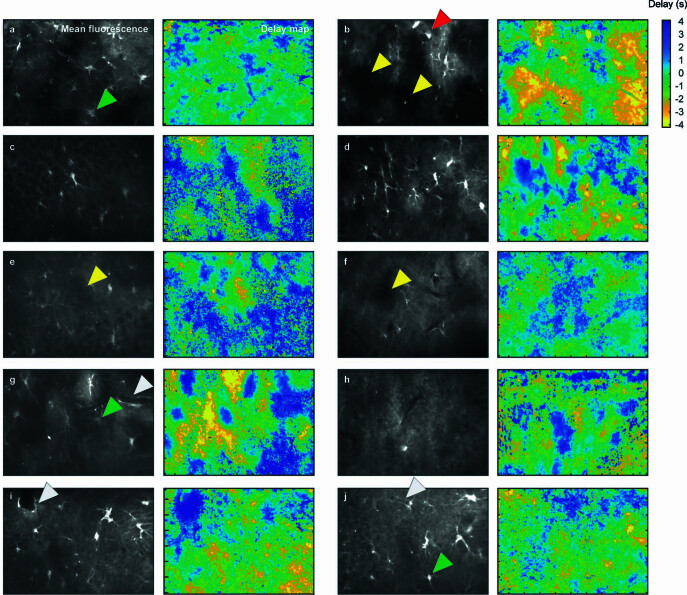
Examples of delay maps. Maps of delays of astrocytic signal with respect to the global mean fluorescence, computed as described in Fig. 5 and Supplementary Fig. 9. Each delay map represents an entire imaging FOV (40× objective, 200 µm side length in x direction). Yellow arrowheads highlight regions that are devoid of somata and thick processes, therefore mostly containing fine gliapil processes. Green arrowheads highlight astrocytic somata that are, unlike other soma examples shown in Fig. 5h and Supplementary Fig. 9, not activated in a delayed manner with respect to the global mean activation. White arrowheads highlight astrocyte processes around blood vessels, exhibiting a delayed activation with respect to the global mean activity. The red arrowhead highlights an ectopically labeled interneuron; such interneuron pixels were blanked for the analysis in Fig. 6.

**Extended Data Fig. 5:**
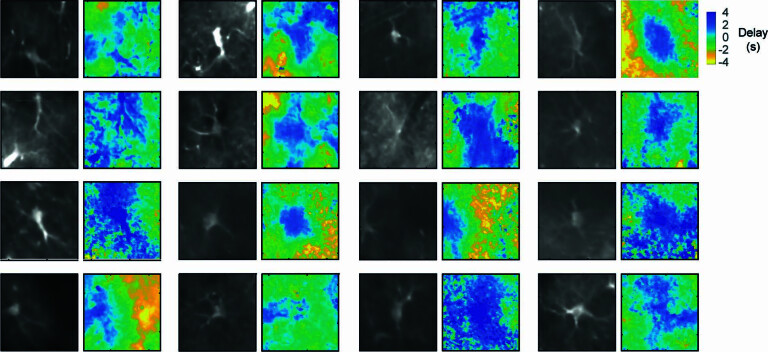
Further examples of isolated astrocytes (fluorescence average, left) together with the local delay maps (right). Extension of Fig. 5h. The color code (as in Fig. 5) indicates the propagation of activity from distal to somatic compartments on a timescale of seconds. The side length of each FOV excerpt is approximately 55 µm.

**Extended Data Fig. 6:**
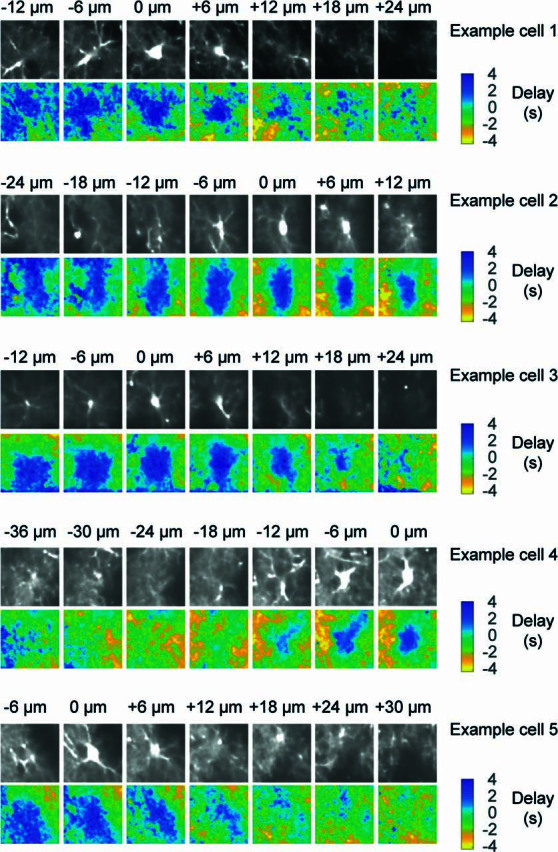
Further examples of 3D delay maps based on dense multi-plane calcium imaging, extension of Fig. 5f. The 3D delay map exhibits the longest positive delay in the imaging plane with the center of the soma. The putative center of the soma is labeled as the ‘0 µm’ imaging plane. The side length of a single tile is approx. 55 µm. Example cells are taken from two imaging sessions in two mice.

To analyze the spatiotemporal astrocytic patterns more accurately, without the bias of manually selected ROIs, we attempted to use the time trace of each pixel to determine a fine-grained delay map. To enable such a precise analysis that is normally prevented by shot noise, we trained and used self-supervised deep networks (Methods) to generate denoised, smooth time courses for individual pixels (Fig. 5e, Supplementary Fig. 9a–h and Supplementary Video 6). The correlation functions of these single-pixel traces revealed a smooth map of delays across the FOVs (Fig. 5g), with features that were obscured in the ROI-based delay map (Fig. 5d). Such delay maps were also visible, albeit less clearly, without denoising (Supplementary Fig. 9i–l). As a striking feature of these pixel-wise delay maps, delays tended to be negative for gliapil regions, whereas they became positive when approaching the astrocytic somata (Fig. 5f–i and Extended Data Figs. 4 and 5). This feature of the delay maps, which we refer to as ‘centripetal integration’, indicates that astrocytic activity, on average, propagates from distal, fine processes to the soma, on a timescale of several seconds. We additionally performed volumetric calcium imaging of multiple closely spaced imaging planes around selected astrocytic somata and computed three-dimensional delay maps, validating this finding in 3D (Fig. 5j and Extended Data Fig. 6). In a few cases, centripetal integration could be visually detected by eye without averaging. For these instances, we confirmed that approaches based on manually drawn ROIs or semi-automatically detected event structures^21^ yielded results consistent with our delay maps based on correlation functions (Supplementary Fig. 10).

To better understand the structural underpinnings of centripetal integration, we analyzed whether propagation occurred diffusely or along visible processes. To this end, we selected the center of identifiable astrocytic somata and defined a 10-µm-diameter ring around this point. Along this ring, regions that contain large processes can be identified based on their increased fluorescence (Fig. 5k). We found that fluorescence along this ring was positively correlated with the delay for almost all astrocytes (Fig. 5k,l; 82 astrocytes from 11 imaging sessions in four mice), suggesting that centripetal propagation indeed proceeds along astrocytic processes. On a side note, we observed that a few astrocytic somata did not exhibit a positive delay, suggesting some heterogeneity (green arrowheads in Extended Data Fig. 5). Furthermore, visual inspection of raw movies showed substantial local activity that cannot be described as propagation from distal to somatic compartments (for example, Supplementary Videos 1, 2 and 6). Therefore, the propagation of activity from distal to somatic compartments dominated the average delay maps but occurred in the presence of other processes.

### Centripetal propagation is conditional on arousal and prior calcium level

**Fig. 6:**
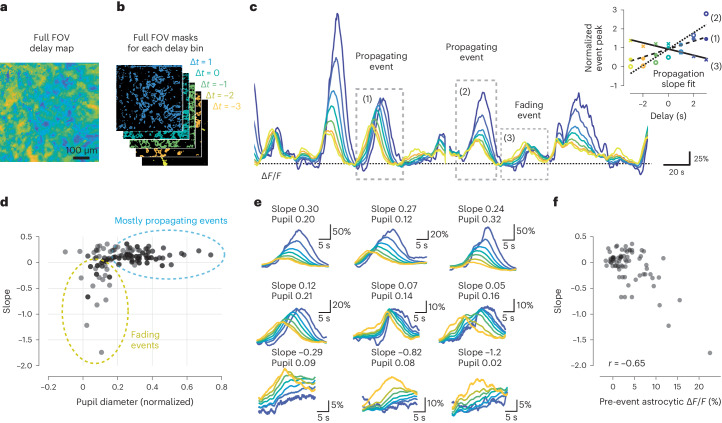
Centripetal propagation of activity in astrocytes is conditional on arousal state and cell-intrinsic calcium signaling history. **a**,**b**, A smoothed delay map of the entire FOV (**a**) is binned according to the delays of each pixel (**b**) (Δ*t* = 1 corresponds to the 0.5 … 1.5 s interval). **c**, The delay bin masks (**b**) are used to extract global astrocytic traces from pixels with a specific delay from the denoised imaging data (color coding). Due to centripetal propagation, yellow traces represent gliapil and blue traces astrocytic somata. Some calcium events propagate to somata (events (1) and (2)), while others do not (event (3)), quantified by a positive or negative propagation slope, respectively (inset). Therefore, centripetally propagating and nonpropagating events can be determined by the slope of peak activity with respect to delay. **d**, Propagation slope of events plotted against the normalized pupil diameter as a proxy for arousal state. Each data point corresponds to a single event. **e**, Example events are plotted in the order of sorted slope values. See Extended Data Fig. 7 for additional example events. **f**, Pre-event astrocytic Δ*F*/*F*, averaged across the 20 s before the event, is negatively correlated with the slope value. Only data points with normalized pupil diameter < 0.2 from **d** were included for **f**. Source data

To compare the time course of individual calcium events in putative distal processes versus somatic regions, we used the delay maps to extract the average time course of all FOV pixels within a specific range of delays (in 1-s bins; Fig. 6a,b). The time course of FOV pixels with negative delays thus reflected activity of distal processes, while the time course of FOV pixels with positive delays reflected activity of somatic regions (Fig. 6c). Previously, we had shown that the mean astrocytic activity was delayed compared to neuronal activity by ~4 s (Fig. 4a). Now, we found that calcium events occurred ~2.5 s later than the average spiking activity of simultaneously recorded pyramidal neurons in most distal processes and ~9 s later in the most central parts (Supplementary Fig. 11). It seems possible that targeted expression in distal compartments would reveal even shorter delays between neuronal and distal astrocytic activity^8^.

**Extended Data Fig. 7:**
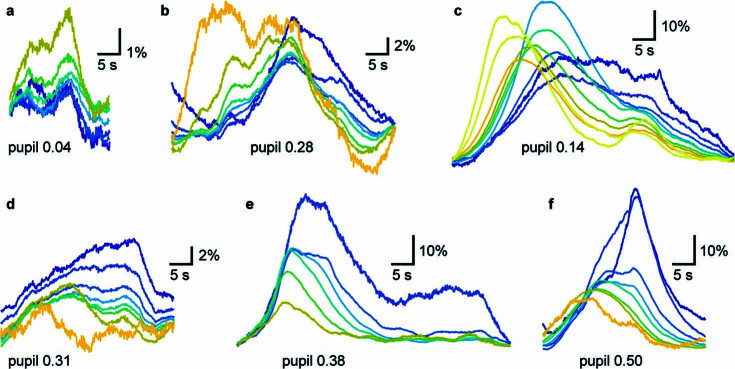
Example events, extension of Fig. 6e. These examples highlight the diversity of astrocytic events that cannot always be well captured as single-phase events. Note the different y-axis scale bars. The x-axis scale bars are identical across events. **a**, Example of a fading event (as defined in Fig. 6) that is barely visible except in the gliapil (yellow). **b**, Biphasic gliapil event. The first gliapil activation (yellow) does not result in centripetal propagation of calcium activity, but a second peak of gliapil activity manages to do so. **c**, Another example of biphasic gliapil activation. The first peak results in centripetal propagation, while the second gliapil peak is only weakly reflected by somatic calcium. **d**, Another example of biphasic gliapil activity. Slow somatic integration barely reflects the faster gliapil fluctuations. **e**, Example of very prominent centripetal propagation, resulting in persistent activation of the somatic region for 10 s of seconds. Note the relatively large pupil diameter. **f**, Similarly prominent centripetal propagation, resulting in a striking display of longer-lasting somatic calcium activity. Note the relatively high pupil value. Source data

Next, we semi-automatically detected and compared individual events (Methods). For many detected events, astrocytic activity reliably propagated from distal processes toward the soma. However, some events started in distal processes but then decayed and failed to activate the cell body (Fig. 6c), indicating a thresholding of centripetal propagation. To quantify whether centripetal propagation occurred or not, we computed a propagation slope for each event, determined by a linear fit of the activity peak of the Δ*F*/*F* trace versus the respective delay. Negative slopes indicate nonpropagating, fading events, whereas neutral or positive slopes indicate centripetally propagating events that activate the soma (Fig. 6c (inset)). Most events exhibited a slope slightly above or around zero, indicating stable propagation toward the center. Some events, however, exhibited a negative slope, reflecting a failure to activate the soma (Fig. 6c–e). These nonpropagating events only occurred when the associated pupil diameter (*z* scored within each session) was small (Fig. 6d) and when the astrocytic calcium signal amplitude was low (Supplementary Fig. 12a). To disentangle long-lasting versus phasic pupil diameter changes, we isolated the phasic component by computing the rectified derivative of pupil diameter as a saliency score. As for the pupil diameter, propagation slopes were negative only for low saliency score values (Supplementary Fig. 12b). These findings indicate that centripetal propagation in astrocytes fails when arousal is low. For very high arousal, on the other hand, we observed events with particularly prominent centripetal propagation. In these cases, centripetal propagation resulted in a nonlinear and persistent activation of the soma that outlasted gliapil activation by 10s of seconds (Fig. 6e and Extended Data Fig. 7e,f). Together, these results suggest that centripetal integration in astrocytes is a nonlinear process that is conditional on the animal’s state and facilitated by high levels of arousal.

We noticed that among events with similar normalized pupil diameter or saliency score, some displayed propagation to the soma (slope > 0) but others did not (slope < 0; Fig. 6d). Previous studies have shown that the strength of astrocytic activation depends on past activity, described as a ‘refractory period’ or cell-intrinsic ‘memory trace’ dependent on intracellular Ca^2+^ store dynamics^15,16,22,23^. Indeed, we found that the propagation slope of events associated with low arousal (normalized pupil diameter < 0.2; Fig. 6d) was negatively correlated with the mean astrocytic Δ*F*/*F* during the 20-s period before these events (Fig. 6f). This analysis shows that centripetal propagation is conditional not only on the animal’s state but also on the prior history of astrocytic activation.

### Variability of centripetal propagation across astrocytes

**Extended Data Fig. 8:**
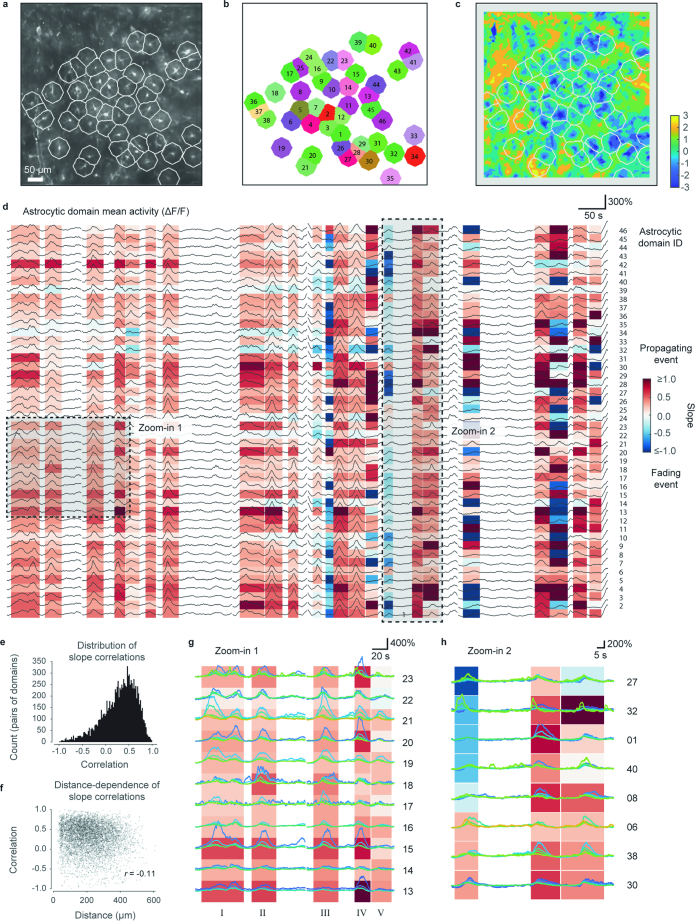
Centripetal propagation can be variable across different putative single astrocytic domains. Manual seeding of the astrocyte cell body centroid with subsequent watershed segmentation was used to create putative astrocytic domains (cell bodies identified based on average fluorescence in (**a**), segmented domains with ID numbers in (**b**)). For each domain, the delay map (**c**) was used to extract delayed traces as shown in Fig. 6a–c, and a slope was fitted to the delayed traces for each detected event and each astrocytic domain, as described in Fig. 6c. Positive slope (blue) indicates an event propagating into the somatic region of the respective domain, a negative slope (red) indicates a fading event (**d**). Overlaid traces display the average activity in the respective domain. **e**, Similarity of slopes across events for pairs of astrocytic domains quantified as correlation. The distribution is centered on positive values, showing that most astrocytic domains follow the propagation/fading of the majority of other domains. **f**, Distance dependence of the pair-wise correlations of propagation slopes from **e**, indicating a weak decay of the correlation with distance. However, low correlations occur also at neighboring domains (distance approx. 20–50 µm), and high correlations at large distances. **g**, Centripetal propagation appears more prominent in some astrocytic domains for some events. Zoom-in #1 from **d** into a subset of events (event numbers in Roman numerals below) and astrocytic domains (numbers on the right side). In addition, delayed traces are shown (color-coding as in Fig. 6; from yellow = distal processes, to blue = astrocytic soma). In some domains, the somatic trace is activated much more than for other domains (for example, domain 15) or for other events in other domains (for example, domain 18 for event II, domain 20 for event IV, and domain 23 for event IV). This observation indicates that the strength of somatic activation by centripetal propagation is variable across astrocytes. **h**, Zoom-in #2 from d, manually re-ordered selection of traces (numbers to the right indicate the putative astrocytic domain). The first event is the event of interest for the purpose of this panel. While the event is globally dominated by fading calcium signals as seen in **d**, calcium activity in some domains clearly reaches the somatic regions (astrocytic domain IDs 30, 38 and 6). This observation suggests that centripetal propagation is a cell-autonomous process that can occur in a subset of astrocytes. Source data

Next, we extended our analysis to individual astrocytes by segmenting distinct astrocytic domains and quantifying centripetal dynamics within individual domains (Methods; Extended Data Fig. 8a–d). Most astrocytic domains in a FOV followed the same pattern for a given event, with either propagating or nonpropagating dynamics. This correlation among domains only weakly depended on interdomain distance (Extended Data Fig. 8e,f). However, the propagation slope in some astrocytic domains was higher than in others during the same event (Extended Data Fig. 8d,g), suggesting that the strength of somatic activation upon centripetal propagation is not binary (propagation versus nonpropagating) but follows a continuous distribution across astrocytes for a given global event. As another interesting and related observation, we noticed some astrocytes that did not follow the global pattern, for example, exhibiting a centripetally propagating event when most other domains did not (Extended Data Fig. 8d,h). These results suggest that conditional centripetal propagation of activity, while mostly synchronized across the population of astrocytes, is a process that has the potential to occur separately in each astrocytic domain.

### Activation of LC reproduces centripetal propagation

**Fig. 7:**
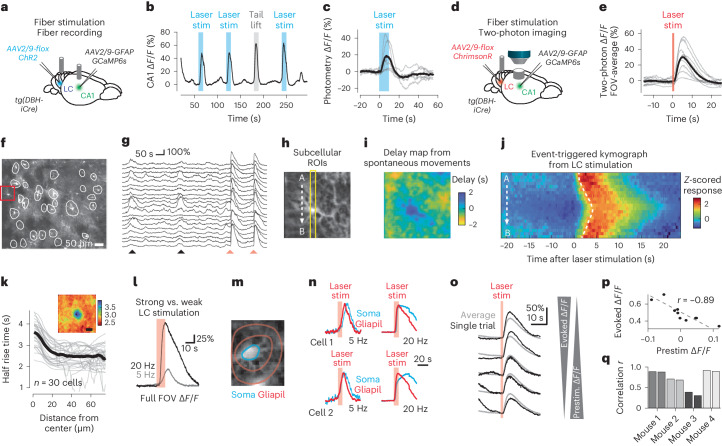
Optogenetic activation of LC triggers centripetal propagation of calcium signals in astrocytes. **a**, Setup for fiber-optic recording of calcium signals in hippocampal astrocytes together with optogenetic LC stimulation. **b**, Example of fiber photometry during LC stimulation (blue) or during tail lift (gray). **c**, Average of opto-evoked ∆*F*/*F* traces (gray, individual mice). **d**, Setup for two-photon calcium imaging of hippocampal astrocytes together with optogenetic LC stimulation. **e**, Average of LC-evoked, FOV-averaged ∆*F*/*F* traces (*n* = 9 sessions across four mice). **f**, Example FOV in the SO with manually labeled ROIs. **g**, ∆*F*/*F* traces corresponding to **f** with astrocytic activity related to movement (black arrowheads) and optogenetic stimulation (red arrowheads). **h**, Zoom-in to single astrocyte (square in **f**). **i**, Delay map for the astrocyte in **h**, computed from denoised imaging data and excluding optogenetic stimulation periods. **j**, Optogenetic stimulation-triggered kymograph along the A→B line in **h**, showing centripetal propagation in a single astrocyte. Average across ten stimulations across 18 min, computed from raw data. Dashed line indicates half-rise time. **k**, Radial plot of the half-rise time of LC-evoked responses as a function of distance from the astrocytic center. Inset, underlying 2D map of half-rise times (scalebar, 20 µm). Averaged across 30 astrocytes with identifiable soma in the imaging plane from four sessions in four mice. **l**, Example of opto-evoked average responses for weak (gray) and strong (black) stimulation. **m**, Manually defined ROIs to separate somatic and gliapil calcium signals. **n**, Examples of persistent activation of soma (blue) but not gliapil (red) for strong but not weaker stimulation in individual astrocytes. Extended Data Fig. 9 for more examples. **o**, Amplitudes of LC-evoked responses decrease for higher prestimulus calcium levels. Example of full-FOV responses for a single imaging session. **p**, Prestimulus ∆*F*/*F* and LC-evoked responses are negatively correlated (quantified from data partially shown in **o**). **q**, The absolute value of the correlation from **p** plotted across mice (two sessions per mouse; statistically higher than for shuffled data with *P* = 2 × 10^−6^, *P* = 0.0007, *P* = 0.0007 and *P* = 0.047 for the four mice; shuffled data were obtained by permuting prestimulus ∆*F*/*F* values; *P* values from 10k shuffles). Source data

Because centripetal propagation was conditional on arousal, we next investigated how arousal is communicated to hippocampal astrocytes. A key brain region to signal arousal throughout the brain is the LC, which previously was shown to activate astrocytes in neocortex by noradrenaline release^10,12^. To understand the role of LC, we used an optogenetic activation approach. First, we expressed the optogenetic activator ChR2 specifically in LC neurons using DBH-iCre mice. In the awake mouse, we stimulated LC with an optical fiber while recording virally induced GCaMP6s bulk fluorescence from hippocampal astrocytes in the ipsilateral CA1 using fiber photometry with a second optical fiber (Fig. 7a). Upon LC stimulation, we observed a striking increase of bulk fluorescence, which was comparable to tail-lift evoked signals (Fig. 7b,c), confirming a role of LC in activating hippocampal astrocytes.

Next, we investigated the subcellular dynamics of LC-driven activation of hippocampal astrocytes by using two-photon calcium imaging in hippocampal CA1 (Fig. 7d–f) combined with fiber-optic optogenetic stimulation of LC with the red-shifted opsin ChrimsonR (virally induced expression in DBH-iCre mice). Consistent with fiber photometry, LC stimulation triggered strong calcium responses across the FOV (Fig. 7e and Supplementary Video 7). Astrocytic calcium signals were increased both in periods of spontaneous movement and during LC stimulation (Fig. 7g). From periods of spontaneous behavior, we replicated our finding of centripetal propagation using delay maps (Fig. 7h,i). For LC stimulation, we observed evoked calcium dynamics that mirrored the spontaneously generated centripetal propagation (stimulation-triggered kymograph for an example neuron shown in Fig. 7j; see also Supplementary Video 8). Quantification revealed that the half-rise time of LC-evoked calcium transients around putative astrocytic somata was larger close to the soma (Fig. 7k; *n* = 30 astrocytes across four mice). These results show that optogenetic LC stimulation replicates not only global astrocytic activation but also centripetal propagation in single astrocytes.

**Extended Data Fig. 9:**
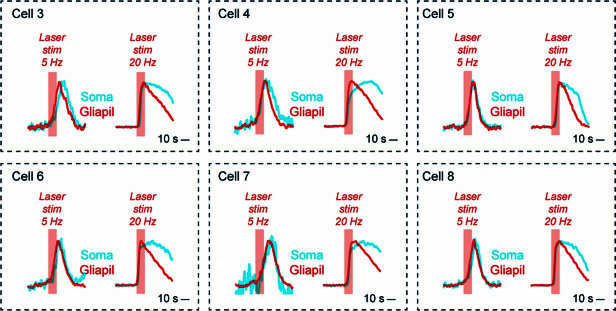
Persistent activation of astrocytic somata but not surrounding gliapil for strong stimulation of locus coeruleus, extension of Fig. 7n. For these astrocytes, somata (blue) were activated shortly after gliapil (red) for weaker stimuli (10 s stimulation, 5 Hz, see Methods for details). For stronger stimulation (10 s stimulation, 20 Hz), somatic calcium signals were not simply a delayed version of gliapil signals but showed a persistent component. Such persistent activation was also observed without optogenetic stimulation during spontaneous behavior (Extended Data Fig. 7e,f). Source data

Astrocytic activation was stronger and faster at higher LC stimulation frequencies (20 Hz versus 5 Hz; Fig. 7l). In addition, we noticed that for 5-Hz stimulation, somatic activation seemed mostly a delayed version of the calcium signal in the gliapil processes, suggesting an approximately linear propagation of calcium signals. For 20-Hz stimulation, however, gliapil calcium signals decayed after stimulation offset, whereas somatic calcium signals persisted much longer (Fig. 7m,n and Extended Data Fig. 9). Interestingly, this nonlinear persistent somatic activation induced by artificial LC stimulation mirrors the persistent somatic signals observed during very high arousal (Extended Data Fig. 8e,f). These results suggest that the strength of LC activation determines the strength and duration of somatic calcium signals upon centripetal propagation.

Additionally, we probed our observation that pre-event calcium concentration levels affect centripetal propagation (Fig. 6f). To this end, we analyzed how LC-evoked ∆*F*/*F* increases were affected by prestimulation ∆*F*/*F* levels. We consistently found across mice that higher prestimulus levels reduced evoked responses, while lower prestimulus levels increased evoked responses (Fig. 7o–q). This finding further supports the idea that calcium signaling during centripetal propagation depends on internal calcium stores that are partially depleted after prominent calcium events.

### Blockade of noradrenalin action reduces centripetal propagation

**Fig. 8:**
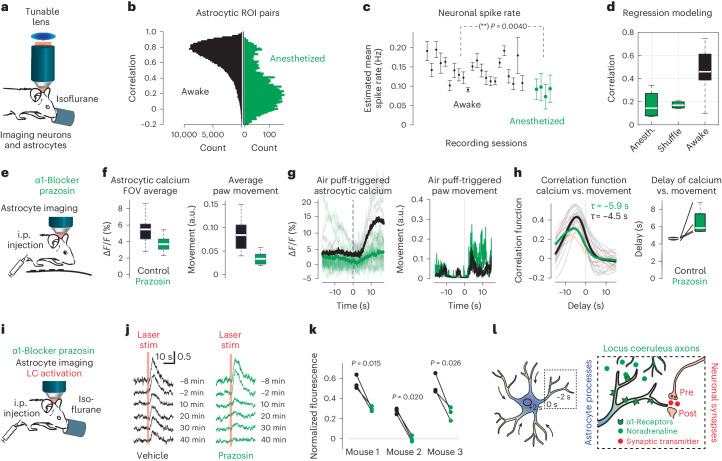
Perturbation of LC-driven noradrenergic signaling impedes global calcium signals and centripetal propagation. **a**, Simultaneous imaging of astrocytes and neurons during isoflurane-induced anesthesia, a state of reduced noradrenergic signals. **b**, Pairwise correlation between astrocytes during awake (corresponds to Fig. 1i) and anesthetized conditions. **c**, Estimated neuronal spike rate (mean ± s.d. across neurons) is slightly decreased for anesthesia compared to wakefulness (*P*_boot_ < 10^−^^4^, hierarchical bootstrapping test; *n* = 22 sessions for wakefulness, *n* = 4 for anesthetized and shuffle). **P = 0.0040. **d**, Global astrocytic activity is well predicted by neuronal activity during wakefulness but not during anesthesia (data from **c**). **e**, Two-photon calcium imaging during behavior after i.p. injection of the α_1_-blocker prazosin. **f**, Prazosin injection reduced average global astrocytic calcium (left; computed with median as *F*_0_ baseline) but also reduced spontaneous movement (right). Nineteen versus eight imaging sessions for control versus prazosin condition in *n* = 4 mice. **g**, Reduced stimulus-evoked calcium signals after prazosin injection (left) despite increased stimulus-triggered movement (right; 17 air puffs for prazosin, six for control condition). **h**, Correlation functions showing that astrocytic activity is more delayed with respect to movement after prazosin injection than after control (left). For paired experiments (animal on first day with saline injection, second day with prazosin condition), delays are increased for each animal (right; *n* = 4 animals; see Supplementary Fig. 13 for per-animal data). **i**, Injection (i.p.) of the α_1_-blocker prazosin with simultaneous astrocytic two-photon imaging and optogenetic LC stimulation during anesthesia. **j**, Example for reduction of opto-evoked calcium signals after injection. The reduction is stronger for prazosin (right) than for saline condition (left). **k**, Reduction of opto-evoked calcium response after prazosin application across three mice. Δ*F*/*F* responses normalized to preinjection levels. Data point triples are taken from timepoints 20, 30 and 40 min after injection; *t* test are applied for these pairs of triples. **l**, Schematic working model of conditional centripetal propagation in astrocytes, controlled by noradrenergic LC signals. For box plots, the median is indicated by the central line; 25th and 75th percentiles are indicated by the box and maximum/minimum values excluding outliers are indicated by the whiskers. Source data

**Extended Data Fig. 10:**
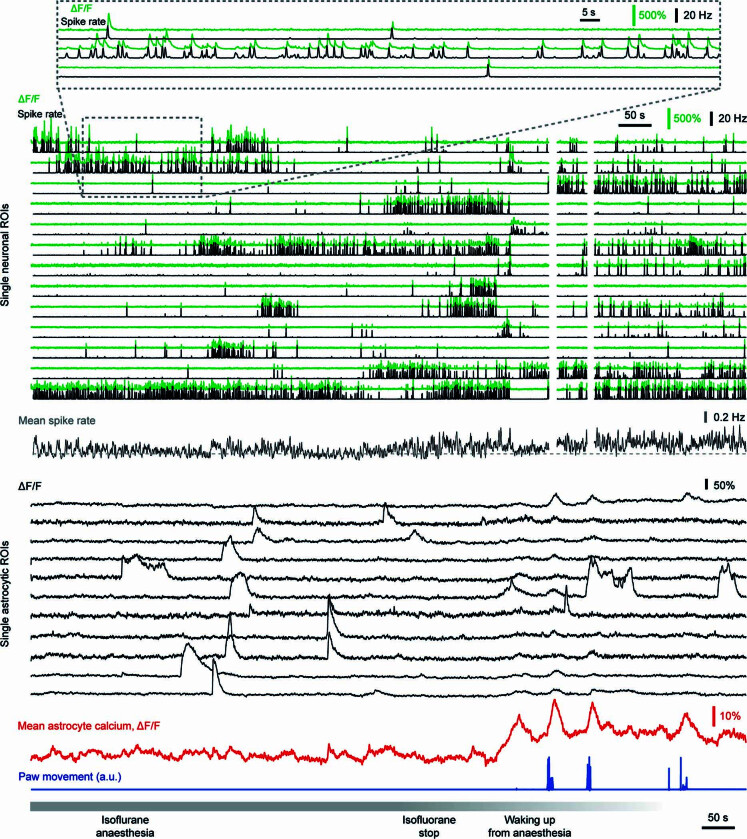
Calcium imaging of astrocytes and pyramidal neurons in CA1 during isoflurane anesthesia. Examples of extracted neuronal ΔF/F traces (green) and associated deconvolved spike rates (black) are shown (see also zoom-in at the top). White blanked time points were discarded due to excessive movement of the brain during waking up. Example astrocytic ΔF/F traces (black) are shown below, highlighting uncoordinated local but no global events before waking up. Waking up (bottom; 1.5% in the beginning, set to 0% at ‘isoflurane stop’) is reflected by small and large paw movements (blue) and resulted in global astrocytic calcium signals (mean astrocytic calcium, red). Source data

We conducted several complementary experiments to block the action of noradrenaline to verify whether we could inactivate astrocytic calcium events and centripetal propagation. First, we investigated astrocytic and neuronal calcium signals during isoflurane anesthesia, a state with reduced noradrenaline release^24^ (Fig. 8a). Astrocytic activity was strikingly reduced during anesthesia, with completely absent global astrocytic events, although some astrocytes still exhibited local events (Fig. 8b and Extended Data Fig. 10). Consistent with recent results in CA1 (ref. ^25^), neuronal spike rates were only slightly reduced (Fig. 8c; 0.14 ± 0.03 Hz versus 0.09 ± 0.01 Hz; *P*_boot_ < 10^−4^). The mean neuronal spike rate, however, failed to explain global astrocytic activity beyond chance level during anesthesia (Fig. 8d; *P* = 0.88, Wilcoxon signed-rank test). These findings are consistent with previous reports of strongly reduced astrocytic calcium levels in the cortex during anesthesia^9,26^. Global astrocytic activity reoccurred, however, when the animal woke up and started to move (Extended Data Fig. 10). These results suggest that awake behavior and the associated neuromodulator release are required for global astrocytic activity.

We next attempted to directly block noradrenaline signaling using the selective α1-adrenergic receptor antagonist prazosin. After i.p. injection of prazosin, calcium signals in the behaving animal were strongly reduced (Fig. 8e,f). However, prazosin exhibited side effects on mouse behavior, in particular a reduction of overall movement (Fig. 8f). Because calcium signals in awake mice are highly correlated with movement (Figs. 3 and 4), it remains unclear whether the reduction of calcium signals was due to α1-receptor blockade or due to reduced movement. For a more controlled experiment, we applied air puffs to induce astrocytic calcium signals and found that responses appeared indeed weaker after prazosin injection compared to control conditions (Δ*F*/*F* increase during a 15-s window upon stimulation: 3.2 ± 1.9% for prazosin, 9.1 ± 4.6% for control; *P* = 0.0087, Mann–Whitney test; Fig. 8g). However, the number of recorded air puffs was low and unequally applied across imaging sessions (total of 17 air puffs in five sessions from four mice for prazosin; 6 air puffs in two sessions from two animals for control) and therefore precludes any strong conclusion.

To further assess the contribution of noradrenaline to centripetal propagation, we reasoned that propagation would be slowed down when the action of noradrenaline was inhibited, similar to our observation of slower centripetal propagation for weaker LC stimulation (Fig. 7l). Such a slowing would be apparent in the delay of astrocytic signals with respect to a reference signal like movement (cf. Fig. 4). We, therefore, analyzed the correlation functions of global astrocytic activity and paw movement. We observed that indeed the correlation functions on average were shifted to longer delays (5.9 s for prazosin versus 4.5 s for control; Fig. 8h) and for each animal (Supplementary Fig. 13; *n* = 4 mice). These results show that astrocytic calcium dynamics during global events were not completely abolished but reduced and slowed down when α1-receptors were inhibited by prazosin.

Finally, we used optogenetic LC stimulation under anesthesia to provide more clearly defined stimulation events. Short calcium imaging segments with LC stimulations were temporally spaced before and after an i.p. injection of prazosin via catheter. LC-evoked responses slightly decreased during the course of anesthesia and also for a vehicle injection (Fig. 8j,k). However, injection of prazosin further decreased evoked responses significantly and consistently across mice (Fig. 8j,k; comparison across three stimulus repetitions 20, 30 and 40 min after injection; for each mouse, *t* test, *P* = 0.026, *P* = 0.015 and *P* = 0.020). As before (Fig. 8e–h), astrocytic calcium responses were reduced but not completely abolished. In conclusion, our inactivation experiments show that awake behavior is necessary for global astrocytic activity and centripetal propagation. Furthermore, blocking α1-receptors reduced global astrocytic signals and slowed down the dynamics that govern centripetal propagation.

## Discussion

We provided evidence that hippocampal astrocytes can be interpreted as slow temporal integrators of salient past events, and we demonstrated that this integration proceeds centripetally—from distal processes to the soma. We further showed that centripetal propagation is conditional both on the state of the astrocyte (past calcium transients) and the state of the animal (level of arousal), and we found that it can be replicated by LC stimulation and impeded by blocking noradrenergic signaling. Together, these findings put forward conditional centripetal propagation as a principle that describes slow spatiotemporal calcium dynamics in astrocytes.

### The global astrocytic mode dominates in awake animals

Recent evidence in cortical and cerebellar brain areas has shown that astrocytic activity in vivo exhibits an FOV-wide, global mode of activation most likely mediated by noradrenergic neuromodulation^5,11,12,15,27^. Here we observed such a global pattern also across hippocampal CA1 layers in awake mice (Fig. 1) but not during isoflurane anesthesia (Fig. 8a–d). In our experiments, astrocytic activation emerged quasi-simultaneously across the FOV (Fig. 5g and Extended Data Fig. 4), consistent with the idea of a global influence converging on astrocytes, and arguing against a dominant role for wave-like propagation of activity within the syncytium of astrocytes through gap junctions (reviewed in ref. ^7^). In line with our finding, noradrenergic axonal terminals in CA1 exhibit highly correlated activity, suggesting a global neuromodulatory effect^28^. Further experiments are needed to investigate global events on a scale beyond our typical FOV sizes (0.6–0.8 mm). Independent of global events, we also observed prominent local activation of individual astrocytes (Fig. 1f,g and Extended Data Fig. 10) as well as rich ongoing subcellular activity (Supplementary Videos 1, 2 and 6), corroborating evidence that localized calcium events in distal processes are a prominent feature of astrocytic dynamics in vivo^7–9,29,30^. Taken together, these experimental findings point to distinct activation modes of astrocytes in vivo that will require further dissection to understand their functions.

### The role of neuromodulation, neuronal activity and movement

Besides noradrenergic neuromodulation, locomotion^12,14–16^ and neuronal activity^4,5,29^ have been shown to affect astrocytic activity. These three processes are tightly entangled: noradrenaline and neuronal activity can act synergistically on astrocytes^12,31^; pupil diameter tracks noradrenergic (and cholinergic) axonal activity with a 1-s delay^19^ and body movements are closely related to pupil diameter changes^32,33^. Here our systematic study of the three factors—local neuronal activity, body movement and pupil diameter—revealed that all factors can explain hippocampal astrocytic signals well if the influence of past events is also taken into account (Figs. 2 and 3). The strong redundancy of these three explanatory variables possibly relates to a common origin of movements, movement-related neuronal activity and neuromodulatory signals in brainstem circuits^34^. Our quantitative description of the temporal sequence of the observed variables (Fig. 4) provides a systematic scaffold to understand and further probe any functional coupling.

Going beyond these observations, our perturbation experiments using optogenetics and pharmacology (Figs. 7 and 8) highlight that noradrenergic signaling through LC is sufficient to induce global astrocytic activity. Under physiological conditions, additional factors might contribute to astrocytic activation, including neuronal activity or other neuromodulatory signals, for example, from cholinergic neurons^35,36^. However, our analyses support the idea of a central role of noradrenergic afferents rather than neuronal activity or movement to gate the emergence of global events.

### A caveat for studying the role of astrocytes during behavior

Our work highlights a potential caveat for future studies of astrocytic activity in vivo. Systems neuroscience has recently become more broadly aware that neuronal signals across the brain might be explained to a substantial amount by spontaneous motor signals^33,37^ or internal states^34^ as confounding variables. Our study suggests that the disentanglement of these processes, which has turned out to be challenging for neurons^34^, is not easier for astrocytes. For example, we show that paw movement is a better predictor of astrocytic activity compared to running speed; therefore, video monitoring of behaviors should be considered more reliable for the observation of confounding motor movements than the measurement of pure displacement (Fig. 3c). In addition, we find that instantaneous measures of coupling fail to capture the delayed relationships of astrocytic signals with movement and neuronal activity (Fig. 3c).

As another caveat, our results suggest that imaging conditions and labeling strategies might have strong and undesired effects on results obtained with astrocytic calcium imaging. For example, astrocytic somata that are slightly out of focus may be more strongly contaminated by the surrounding gliapil, resulting in temporally shifted calcium transients (Fig. 5a–d). Similar concerns apply to imaging conditions where resolution is degraded, resulting in the mixing of somatic and gliapil signals. In view of these challenges, previous studies claiming specific computational roles of hippocampal astrocytes, for example, for reward or place encoding^3,6^, would probably benefit from careful controls for confounding variables, in particular those that are shifted in time by several seconds. Specifically, our study provides strong evidence that astrocytic activity in the hippocampus is more parsimoniously explained as a response to past arousing events rather than reflecting an expectation of future spatial location as recently suggested^6^.

### Correlation functions to extract astrocytic activity modes

Recent years have seen a surge of tools specifically designed for the analysis of astrocytic calcium imaging. However, these tools are based either on discrete spatial ROIs^20,38,39^ or on discrete events^21,40^, in both cases requiring a definition of events via arbitrary criteria. Here we used a method without such a requirement by computing pixel-wise delay maps with respect to the global astrocytic activity as a reference. This analysis takes advantage of the averaging power of correlation functions and projects the extracted delay onto the anatomical map, pixel by pixel (Fig. 5). Moreover, early and late temporal components can be extracted from the delay maps and further studied (Fig. 6). This workflow is generally applicable for the unbiased extraction of average spatiotemporal activity patterns from calcium movies. We provide a well-documented demo code on GitHub in both MATLAB and Python to facilitate the adoption of our method^41^.

### Conditional centripetal integration in astrocytes

The most striking feature of hippocampal astrocytes is their star-shaped morphology. Here we provide evidence that the soma as the center of this ‘star’ acts as an integration hub that is activated upon salient events. First, we could describe astrocytic activation well by a leaky integration differential equation (Fig. 3j,k), revealing that neuronal activity, movement and pupil changes precede astrocytic activity in a sequence with consistent delays (Fig. 4). Our results are consistent with previous studies investigating subsets of these observables^5,11,12,15,16,31^. Second, we show that the temporal integration is accompanied by a spatial integration with activity propagating from distal astrocytic processes to the soma on a timescale of several seconds (Fig. 5). Consistent with the idea of earlier distal activations, recent studies have found low-latency activation of astrocytes in fine distal processes^8,9^. It has been hypothesized^42^ and shown in hippocampal slices that small calcium events are often restricted to fine processes but expand for stronger stimuli^43^, and can invade the somatic region^9^. Our results extend these findings and establish centripetal propagation as an important feature of calcium dynamics in hippocampal astrocytes during behavior. Furthermore, our analyses highlight that centripetal integration is a nonlinear process that can be restricted to a subset of astrocytes (Extended Data Fig. 8) and that can induce long-lasting somatic activation in individual astrocytes (Fig. 7m,n and Extended Data Fig. 9). Interestingly, a recent study has observed centripetal propagation in cortical brain regions^44^; hence, it is likely that this phenomenon is not restricted to hippocampus.

Our analyses and optogenetic experiments indicate that centripetal propagation is conditional on at least the following two factors: first, it is facilitated by high LC activity (Fig. 7), which reflects higher levels of arousal (Fig. 6); in addition, it is impeded for already elevated calcium concentrations (Figs. 6f and 7o–q). Previous in vivo experiments showed that calcium events in late- but not fast-responding astrocytic regions depend on both IP_3_ (refs. ^8,20^) and noradrenergic signaling^8^. It is therefore a reasonable hypothesis that calcium signals in distal astrocytic processes are driven by local synaptic activity (largely independent of IP_3_ and noradrenaline) and then propagate to the soma in an IP_3_-dependent manner only when saliency is communicated through noradrenergic signals. Our results therefore suggest that the astrocytic soma operates as a computational unit and acts as an integrator of past saliency.

### A role for astrocytes to slowly process past events

In typical neurons, information from dendrites is integrated at the soma, generating output that is further conveyed via action potentials. Our finding of centripetal integration in astrocytes therefore raises the question about the potential output generated upon their somatic activation. A possible astrocytic output signal that warrants further investigation in the context of centripetal integration is gliotransmission, the astrocytic release of GABA^45^, glutamate^46^, d-serine^35,47^, lactate^48^ or other transmitters. The function of such an output remains unclear but is constrained by the properties of centripetal integration. First, global astrocytic activity can be described as a temporal integration of past salient events (Figs. 3 and 4). Second, centripetal propagation of astrocytic activity is closely associated with arousal, and the LC is a key regulator of arousal (Figs. 3, 7 and 8). Third, centripetal propagation acts on a timescale of seconds rather than milliseconds (Figs. 4 and 5), arguing against low-latency contributions to neural computations and rather for a role in processes that act on longer timescales. Modulation of neuronal plasticity is a plausible candidate for such a process because it acts on behavioral timescales^49^ and takes place upon salient events. Such modulation has been studied for astrocytes in the hippocampus and other regions in vitro^35,47,50,51^ and more recently in vivo^36,52–54^, with gliotransmission as a key factor linking astrocytes to neuronal plasticity^42,55^. It remains to be discovered how somatic astrocytic activity could translate into potentiation or depression of specific synapses. However, centripetal integration of past events defines a plausible candidate mechanism of how such conditional output of astrocytes could be orchestrated on a behavioral timescale.

## Methods

### Animals and surgery

All experimental procedures were carried out in accordance with the guidelines of the Federal Veterinary Office of Switzerland and were approved by the Cantonal Veterinary Office in Zurich. All animals were group-housed on a reversed 12-h light/12-h dark cycle at a temperature between 21 °C and 23 °C and humidity between 55% and 60%. We used adult male and female 4- to 6-month-old C57BL/6-Thy1-GCaMP6f (GP5.17; ref. ^56^), which express GCaMP6f in a subset of pyramidal neurons in hippocampal CA1, C57BL/6-Tg(Dbh-iCre)1Gsc mice^57^, which express Cre in noradrenergic neurons, and wild-type C57BL/6 mice. Mice were provided with analgesia (Metacam, 5 mg kg^−1^ bodyweight and Buprenorphine, 0.1 mg kg^−1^, s.c.) before surgery. Anesthesia was induced using isoflurane (5% in O_2_ for induction, 1–2% for maintenance during surgery), and the body temperature was maintained at 35–37 °C using a heating pad. For surgeries, shaving cream was applied to the dorsal head above the brain, and an incision was made into the skin after local application of lidocaine. To induce expression of GCaMP6s in astrocytes, an injection of adeno-associated virus (AAV) based on the human glial fibrillary acidic protein (GFAP) promoter fragment gfaABC1D (ca. 200 nl of ssAAV9/2-hGFAP-hHBbI/E-GCaMP6s-bGHp(A), titer 1.0 × 10^13^ vg ml^−1^; Viral Vector Facility, University of Zurich) was made in hippocampal CA1 (coordinates: anterior-posterior (AP) −2.0 mm; medial-lateral (ML) −1.5 mm from bregma and dorsal-ventral (DV) −1.3 from the surface of the dura). The injection pipette was left in place after injection for at least 5 min to prevent reflux. The skin was closed with a suture and reopened after 2 weeks to implant the hippocampal window, as described previously by others^58^ and ourselves^59,60^. Briefly, to expose the brain, a 3-mm diameter craniotomy centered at the previous injection site was drilled. For attachment, two layers of light-curing adhesive (iBond Total Etch, Kulzer) were applied to the skull, followed by a ring of dental cement (Charisma, Kulzer) to prevent overgrowth with skin. A 3-mm diameter biopsy punch (BP-30F, KAI) was inserted until it reached the corpus callosum and left in place for >5 min to stop bleeding. With a flatly cut-off injection cannula (Sterican 27G, Braun) connected to a vacuum pump, the cortex was carefully removed until the stripes of the corpus callosum became visible. The corpus callosum, different from previous studies targeting deeper regions^59^, was left intact. Bleedings were stopped with absorbent swabs (Sugi, Kettenbach) and hemostatic sponges (Spongostan, Ethicon). Then, a cylindrical metal cannula (diameter = 3 mm and height = 1.2–1.3 mm) attached with dental cement to a 0.17-mm thick coverslip (diameter = 3 mm) was inserted into the cavity and fine-positioned with a glass capillary attached to the stereotaxic frame. When no further bleeding occurred, the hippocampal window was fixed in place using dental cement (Tetric EvoFlow, Ivoclar). Finally, tissue glue (Vetbond, 3M) was used to connect the animal’s skin with the ring of dental cement. A head bar was attached to the Charisma ring using dental cement (Tetric EvoFlow). After surgery, animals were monitored for 3 days with the application of antibiotics (2.5% Baytril in drinking water; Vetpharm) and analgesics (Metacam; 5 mg kg^−1^, s.c.) were administered when necessary. Behavioral training started 2 weeks after surgery. Calcium imaging was performed 2–3 weeks after the start of behavioral training.

For optogenetic experiments in DBH-iCre mice, we followed procedures as previously described^61^. Briefly, a small hole was drilled at AP −5.4 mm and ML −0.9 mm relative to bregma. Mice were then injected unilaterally (AP, −5.4 mm; ML, −0.9 mm and DV, −3.8 mm) with 1 µl of an AAV construct carrying the optogenetic actuators ChR2 or ChrimsonR (ssAAV-5/2-hEF1α-dloxhChR2(H134R)_EYFP(rev)-dlox-WPRE-hGHp(A) or ssAAV-5/2-hEF1α/hTLV1-dloxChrimsonR_tdTomato(rev)-dlox-WPRE-bGHp(A); Viral Vector Facility, University of Zurich) using a pneumatic injector (Narishige, IM-11-2) and calibrated microcapillaries (Sigma-Aldrich, P0549). During the same surgery, 400 nl of astrocyte-specific GCaMP6s-inducing AAV was injected into hippocampal CA1 as described above. For fiber photometry experiments (Fig. 7a–c), optical fibers were implanted 200 μm above the injection coordinates of LC and hippocampus (diameter = 200 µm and NA = 0.37; Neurophotometrics). For LC stimulation combined with two-photon imaging (Fig. 7d–q), an angled optical fiber was implanted (low profile, angle = 90°, diameter = 200 μm and NA = 0.66; Doric Lenses). Optical fibers were glued to the skull using a bonding agent (Etch glue; Heraeus Kulzer GmbH) and a UV-curable dental composite (Permaplast, LH flow; M+W Dental). After 3 weeks, pupillometry was performed as described before to validate the functional expression of the actuator^62^. For animals that showed pupil responses upon LC stimulation, hippocampal windows were implanted as described above.

### Two-photon microscopy

A custom-built two-photon microscope was used to monitor calcium signals in astrocytes and neurons in either single or multiple layers of CA1. A femtosecond-pulsed laser (MaiTai, Spectra-Physics; 911 nm; power below the objective, 20–40 mW) was sent through a scan engine consisting of an 8-kHz resonant scanner (Cambridge Technology), a ×2 magnifying relay lens system and a slow galvo scanner (Cambridge Technology, 6215H), to scan lens (Sill Optics, S4LFT0089/98) and tube lens (Ploessl lens consisting of two 400-mm focal length achromatic doublet lenses; Thorlabs, AC508-400-AB) before entering the objective’s back aperture. Either a ×16 (CFI75 LWD 16X W, numerical aperture NA = 0.8, working distance WD = 3.0 mm; Nikon) or a ×40 objective (CFI Apo NIR 40X W, NA = 0.8, WD = 3.5 mm; Nikon) was used for calcium imaging. The ×16 objective provided a larger FOV (600 µm side length), but the back aperture was slightly underfilled, resulting in an axial resolution of 4–5 µm (full width at half maximum, FWHM). The ×40 objective allowed us to overfill the back aperture, resulting in an improved axial resolution of 2–3 µm (FWHM) at the cost of a reduced FOV (200 µm side length). The ×40 objective configuration also enabled the use of a small-aperture tunable lens (Optotune, EL-10-30-C) together with an offset lens (*f* = −100 mm) just before the back focal plane for fast z-scanning over up to 300 µm as described previously^63^. For experiments with simultaneous photostimulation of LC, a ×10 long working distance water immersion objective was used (Olympus XLPLN10XSVMP, NA = 0.6, WD = 8 mm) to avoid spatial constraints with the stimulation fiber below the objective, resulting in a larger FOV (600–1,000 µm). Single-plane imaging was performed at a rate of 30.88 Hz (512 × 622 pixels). Volumetric rates were reduced accordingly for dual-plane (15.44 Hz), triple-plane imaging (10.29 Hz) and imaging across seven planes (4.41 Hz). Scanning and data acquisition were controlled with custom-written software programmed in C++ (http://rkscope.sourceforge.net/, ref. ^64^).

### Fiber photometry

GCaMP6s signals were recorded using a commercially available photometry system (Neurophotometrics, Model FP3002) controlled via the open-source software Bonsai (2.6.2 version). The implanted fiber was attached to a prebleached recording patch cord (diameter 200 μm and NA = 0.39; Doric Lenses). Two light-emitting diodes (LEDs) were used to deliver interleaved excitation light—a 470 nm LED for recording GCaMP-dependent fluorescence signal (*F*^470^) and a 415 nm LED for GCaMP-independent fluorescence signals (*F*^415^) with 60 Hz for each channel. Excitation power at the fiber tip was set to 25–35 μW. Analysis of raw photometry data was performed using a custom-written MATLAB script as described previously^61^. First, to filter high-frequency noise (above 1 Hz), a lowpass filter was applied to both signals (*F*^470^ and *F*^415^). Next, to correct photobleaching, the baseline fluorescence was calculated as a linear fit of the filtered *F*^415^ signal to the level of the *F*^470^ signal during the 5-s baseline window preceding each LC stimulation. This rescaled *F*^415^ signal is termed *F*^415^_baseline-fit_. Finally, GCaMP signals were expressed as the Δ*F*/*F* value:

1 $$\Delta {{F}}/{{F}}=100\times \left({{{F}}}^{\;470}({{t}}){{\mbox{-}}}{{{F}}}_{{\rm{baseline}}\,{\rm{fit}}}^{\;415}({{t}})\right)/{{{F}}}_{{\rm{baseline}}\; {\rm{fit}}}^{\;415}({{t}})$$

where *F*^470^(*t*) signifies the filtered fluorescence value at each timepoint *t* across the recording and *F*^415^_baseline-fit_(*t*) denotes the value of the fitted 415 nm signal at the timepoint *t*. The final Δ*F*/*F* signal was smoothed with a 100-point moving mean filter.

### Optogenetic stimulation

LC was optogenetically stimulated via ChR2 (fiber photometry; Fig. 7a–c) or ChrimsonR (two-photon imaging; Fig. 7d–q) using a 473-nm or 635-nm laser with a fiber output power of 10 mW when illuminating continuously. Stimulation protocols followed previous work to provide stimulation similar to natural LC activity during arousal^61^. In fiber photometry experiments, tail lifts were performed as previously described^65^. Long stimulations (10-s duration) were used to provide consistency with previous work^61^. Short stimulations (≤1 s) were used to provide clearly defined phasic stimuli, which more easily enabled stimulus-triggered analysis. For all stimulation protocols, 10-ms pulses of light were applied, but at different repetition frequencies. For experiments described in Figs. 7f–m,q–s and 8i–k, we used a 1-s stimulation with a pulse repetition frequency of 15 Hz (average power 1.5 mW). For experiments described in Fig. 7a–c,n–p, we used a 10-s stimulation with a pulse repetition frequency of 5 Hz or 20 Hz (average power, 0.5 and 2.0 mW).

### Behavioral setup

The treadmill consisted of two custom-designed lightweight wheels, one of which was attached to a rotary encoder (4-mm shaft optical rotary encoder; Phidgets) to measure the locomotion of the animal. A 130 cm long and 5 cm wide velvet belt (McMaster-Carr, 88015K1) was equipped with sensory landmarks consisting of self-sticking elements, velcro strips and hot glue. A metal tape attached to a single location on the back side of the belt was used as a reflector for an infrared (IR) sensor to provide a spatial reference to track the location of the animal. IR light (Thorlabs, LIU850A) together with a camera (DMK23UP1300, The Imaging Source, recording at 30 Hz; 16-mm EFL objective MVL16M23; Thorlabs) was used to monitor the animal’s behavior during the experiment. In a subset of experiment sessions (33 of 42), a UV LED (Thorlabs, LED370E) was directed toward the right eye of the animal, resulting in a less dilated pupil to enable pupil segmentation. Sweetened water rewards (30% sugar) were provided through a metal lick spout at a specific location of the belt. Reward delivery was controlled by a solenoid valve (VDW22JA, SMC) that was gated by a relay circuit (Sertronics Relais Module, Digitech). Mice were free to consume the reward, immediately after reward delivery or later. If mice did not retrieve a reward for 50 s through running, a spontaneous reward was delivered. Reward deliveries were automatically recorded, and reward consumption (first contact of the mouse’s tongue with the water drop) was manually detected from video monitoring. The time of reward consumption was used for analyses in Extended Data Fig. 2a,b. In a subset of experiments, brief air puffs to the left side of the animal’s face were provided randomly and rarely (maximally once per minute). To enable the disentanglement of arousal generated by air puffs and movement, air puffs were only applied when the animal was not running for ≥10 s.

The behavioral setup was controlled using custom-written Python code, which controlled valves, camera triggers and microscope acquisition start, and recorded the position of the rotary encoder, the IR sensor and behavioral events like air puffs or water rewards. The temporal offset and relative frame rate of the camera with respect to two-photon scanning were calibrated separately, with both camera and two-photon software recording the same signal of a flashing light.

### Behavior training and imaging experiments

One week before experiments, drinking water of mice was supplemented with citric acid (2% of volume) to motivate the mice during the running task^66^. Mice were handled for 15–20 min per day for 3 days. Afterward, they were accustomed to the behavioral setup and the lick spout. For the next 3–7 workdays, mice were trained to be head-fixed on the treadmill. When animals readily ran on the treadmill and consumed sugar water rewards for 15–20 min, imaging experiments were performed.

A behavioral session lasted for 15–35 min and consisted of 140-s-long segments, spaced by breaks of 5–20 s due to the microscope software. The imaging plane was chosen to lay in the central part of stratum oriens of CA1 and to contain visible astrocytic somata if possible. For dual-plane imaging of neurons and astrocytes, the first imaging plane was centered at the pyramidal cell layer of CA1, and the second imaging plane 60–90 µm more dorsally. For triple-layer imaging of astrocytes, imaging planes were spaced ~70 to 80 µm from each other, with the central plane residing in the pyramidal cell layer. For triple-plane imaging during anesthesia, two imaging planes spaced by 10–15 µm were positioned in the pyramidal layer of CA1 such that neurons were not sampled twice and the third plane 60–90 µm more dorsally. For volumetric imaging of astrocytes, the seven imaging planes around an astrocyte were spaced by 6 µm between adjacent imaging planes. Different sessions were performed on different days, and the FOV was changed between days.

Data collection and analysis were not performed blind to the conditions of the experiments. Stimuli (air puffs, spontaneous rewards) were given randomly but adapted to the behavior of the animal. For example, no spontaneous rewards were given when the water was not consumed, and no air puffs were given when the mouse appeared highly stressed by the stimulus.

### Pharmacology

Prazosin (Tocris, 0623) was dissolved in distilled water (10 mg in 100 ml Millipore). For experiments described in Fig. 8e–h, mice were lightly anesthetized in isoflurane, i.p.-injected with 10 µl g^−1^ (solution/bodyweight) and then were allowed to wake up from anesthesia without delay. Anesthesia during injection was applied to facilitate procedures and to reduce stress by injection. Experiments started 20–25 min after injection and lasted 15–25 min. For experiments described in Fig. 8i–k, animals were anesthetized (5% isofluorane) and then quickly transferred to the head-fixation setup, where anesthesia was continued (2% and 1.5% isoflurane for male and female mice, respectively). A catheter consisting of the tip of an insulin needle (Braun, Omnican 50 LDS) was inserted for i.p. injection via a syringe. After baseline recording, i.p. injection was performed, and optogenetic stimuli were applied together with calcium imaging every 10 min after the timepoint of injection. For control, the procedure was repeated with distilled water instead of prazosin solution.

### Histology

After completion of calcium imaging experiments (~2 months after the start of behavioral experiments), animals were administered a lethal dose of pentobarbital (Ekonarcon, Streuli) and transcardially perfused with 0.1 M phosphate buffered saline (PBS) followed by 4% paraformaldehyde (in 0.1 M PBS). For histology experiments to check colocalization of GCaMP and GFAP (Extended Data Fig. 1e–g), 60-µm-thick coronal brain sections were stained with the nuclear stain DAPI and anti-GFAP (primary AB rabbit-anti-GFAP 1:1,000, secondary AB anti-rabbit with Cy3 1:250) and imaged with a confocal laser-scanning microscope (Olympus FV1000). Three separate channels recorded DAPI, intrinsic GCaMP6s and antibody staining with Cy3. For experiments to compare GFAP levels in injected versus noninjected hemispheres of the same animal (Extended Data Fig. 1a–d), the same histology procedure was applied. Care was taken to apply the same imaging power, zoom settings and imaging depth below the surface for ipsilateral and contralateral imaging sites. For the quantification of mean fluorescence and area fill fraction, we followed existing standard protocols, with the same threshold used to compute the fill fraction for both hemispheres for each slice^67^.

### Calcium imaging postprocessing

Calcium imaging movies were spatially resampled to remove the distortion induced by resonant scanning. Then, rigid movement correction in the *xy*-plane was applied^68^. Analyses in Fig. 1 were based on manually defined ROIs; analyses in Figs. 3, 4 and 5 were based on the global astrocytic activity, which we define as the average Δ*F*/F signal across the FOV; and analyses in Figs. 6 and 7 were based on pixel-based activity traces (described below). Active ROIs were extracted manually with a previously described toolbox (https://github.com/PTRRupprecht/Drawing-ROIs-without-GUI, ref. ^69^), based on the mean fluorescence and the map of local correlations^70^.

For neuronal imaging data, both active and inactive neuronal somata were included for an unbiased estimate of neuronal spike rates. For experiments with expression in both neurons (GCaMP6f) and astrocytes (GCaMP6s), bleedthrough of the gliapil signal in the pyramidal layer resulted in a slow contaminating signal superimposed onto the neuronal signals. We used the mean fluorescence in a 15-pixel-wide surround region of the respective neuronal ROI to linearly unmix the astrocytic contamination (Supplementary Fig. 2). Pixels of other neuronal ROIs were not included in the surround ROI. Next, deconvolution of the neuronal traces with a supervised deep network (CASCADE; ref. ^18^) was performed in Python, suppressing shot noise^18^ but also discarding nonneuronal signals like slow components stemming from astrocytes (Supplementary Fig. 2).

For astrocytic imaging data, all clearly visible astrocytic cell bodies were selected as ROIs, as well as active gliapil regions with a spatially coherent response, as seen by the map of local correlations (Supplementary Fig. 1). Processes that were putatively part of a single astrocyte due to correlated activity, as seen by the map of local correlations, were included in a single ROI. Contamination of GCaMP6s signals through neuronal signals (GCaMP6f) for dual-layer imaging was negligible, primarily due to the spatial separation of pyramidal neurons from the astrocytes in the SO. The absence of contamination is evidenced by the correlation function between neuronal and astrocytic activity (Fig. 4a), which does not exhibit a peak at *t* = 0 s. Due to the known leakiness of the GFAP promotor, also a sparse set of interneurons was labeled with GCaMP6s. These rare cells (0.7 ± 0.6 cells per FOV%; mean ± s.d. across 19 FOVs from three animals) were identified based on their distinct morphology and based on their quickly fluctuating signals, which were clearly distinct from astrocytic signals (Supplementary Fig. 4a–c). We computed the fraction of pixels in a FOV covered by interneurons and found it to be much smaller than 1% (0.13 ± 0.19%; mean ± s.d. across 19 FOVs from three animals).

Residual movement artifacts along the z-direction were visually detected from the extracted temporal traces as events of both correlated and anticorrelated changes of fluorescence across a majority of the FOV. These events were inspected in the raw movies and blanked for further analysis. Mice for which such strong motion artifacts occurred regularly were not used for experiments.

### Behavioral monitoring postprocessing

The behavioral video was used to extract several behavioral features. The correlation between subsequent frames of subvideos (mouth or front paws region) was used to compute movement. The measured metric (1 − correlation) was scaled by the within-session maximum. To extract higher-dimensional face movements, we used a singular value component analysis of face movements^33^ and used a combination of these components to explain astrocytic signals (Supplementary Fig. 4).

The pupil appeared bright in the behavioral camera video due to infrared laser light exiting the pupil. The equivalent diameter was computed from the area of the pupil, which was obtained from a segmentation of the pupil with standard image processing methods. Briefly, the brightest round object in the ROI covering the eye was extracted from the binarized image using the regionprops() function in MATLAB (MathWorks). Dark spots inside the segmented pupil due to reflections were filled, and connections of the segmented pupil to the bright upper eyelid were removed by repeated binary erosion and dilation of the segmented pupil. Occasional winking events were manually detected from the extracted pupil diameter traces, confirmed by video inspection, and automatically replaced by values obtained via linear interpolation. The difference between illumination periodicity (from the imaging system, 30.88 Hz) and the video recording rate (30 Hz) resulted in a beating pattern of 0.88 Hz that was also visible in the extracted pupil signal. A template filter based on the 0.88 Hz periodicity was used to remove this illumination-induced component from the extracted pupil diameter. For the analyses in Figs. 3 and 4, the absolute pupil diameter was used. For analyses associated with Fig. 6, to allow for pooling of pupil diameter values across sessions with variable lighting conditions, pupil diameters were *z*-scored for each behavioral session.

Similarly, licking was quantified by classical segmentation methods that detected the presence of a bright and large object close to the lick spout (the tongue). The final output was binarized (licking versus nonlicking) based on a threshold of detected object size. The threshold for lick detection was adjusted manually for each session and for each mouse while inspecting the resulting segmentation together with the raw behavioral movies.

The absolute position of the mouse on the treadmill belt was computed using the run speed recorded with the rotary encoder as a relative position signal and the analog IR diode output, which increased when a reflective tape at the backside of the belt went past the diode, as an absolute position signal.

### Modeling global astrocytic activity with dilated linear regression

To model global astrocytic activity as a function of other variables (for example, paw movement, pupil diameter or location), we used a dilated variant of linear temporal regression. Specifically, we averaged timepoints of the regressor around the to-be-regressed timepoint into bins that exponentially increased their width with the temporal distance from the current timepoint. Therefore, the first bin was 1 frame in width, the second 2 frames, the third 4 frames, the fourth 8 frames, and so on, resulting in an effective time window of ±17 s (±512 timepoints sampled at 30 Hz). This ‘dilated linear regression’ is inspired by ‘dilated convolutions’ used for signal processing and deep neuronal networks^71,72^ and serves to reduce the number of regressors while providing a multiscale representation of the regressor variable. Such a reduction of the number of regressors is necessary to avoid overfitting. Overfitting is a problem when regressing global astrocytic signals, which provide—due to their slowly changing nature—only relatively few statistically independent data points.

The vector of such dilated regressors was used to linearly regress the observed global astrocytic activity with the *glmfit()* function in MATLAB. Performance was evaluated on fivefold cross-validated subsets within each recorded session to exclude overfitting. Performance was measured using the correlation between predicted and true signals across the 5 cross-validated segments of the entire session.

### Modeling global astrocytic activity with a linear differential equation

A standard leaky-integrator differential equation of the form

2 $$\tau \frac{{dA}(t)}{{dt}}=-A\left(t\right)+I(t)$$

was implemented, with the integration time constant *τ*, the astrocytic activity *A*(*t*) and the regressor input *I*(*t*). Fitting was performed by grid search of the parameter *τ* and by evaluating the correlation of the recorded with the simulated signal. Because correlation is not affected by the scaling signals, a scaling factor of the input regressor *I*(*t*) was not necessary. For the evaluation, the first timepoints until *t* = 2*τ* were excluded to avoid the influence of the initial conditions during the simulation.

### Self-supervised denoising of calcium movies

Due to the sensitivity of astrocytes to laser-induced heating^73^, we limited the imaging laser power to moderate values, at the cost of a decreased signal-to-noise ratio. To enable analyses with single-pixel precision despite noisy pixel traces, we used algorithms for self-supervised denoising based on deep networks^74,75^, implemented in the Python scripts of DeepInterpolation^76^. This implementation uses the pixels of the 30 frames before and after the current frame to denoise the pixels of the current frame. More precisely, the algorithm infers for each pixel the value that is most likely, based on its spatiotemporal surrounding pixels and based on the priors of the networks. To adapt the network before our imaging data, we retrained the deep network from scratch for each analyzed session with 10,000 frames of the recording and then ran the trained network on all imaging frames of the respective session. To avoid movement artifacts through brain motion, we applied an algorithm for piecewise rigid motion correction on the denoised data^77^.

### Delay maps of astrocytic activity

We used the global astrocytic activity (average across the entire FOV) as a global reference to compute the average delay of each pixel’s signal. To extract the average delay, we computed the correlation function between the reference signal and the pixel’s time trace, which was extracted from the denoised movie (described above). The correlation function was normalized such that the zero-lag component was Pearson’s correlation coefficient. Next, the correlation function was smoothed with a 0.5-s window filter, and the delay was determined by taking the maximum of the correlation function in a −10 … +10 s window. This procedure was repeated for each pixel, resulting in a map of delays. Code in both MATLAB and Python to compute delay maps from raw or denoised data is provided, together with sample data, and documented at https://github.com/HelmchenLabSoftware/Centripetal_propagation_astrocytes.

Delay maps were computed for each 140-s segment in a session, and per-segment maps were combined into a session map using weighted averaging, with mean astrocytic Δ*F*/*F* of the entire FOV within each segment used as weights for averaging. Delay maps were computed for a total of 12 selected sessions that fulfilled the following criteria: (1) a sufficient amount of global astrocytic activity. For example, sessions with mice that barely moved during the sessions did not exhibit sufficient global astrocytic dynamics. (2) Clearly visible astrocytic somata. In some sessions, especially when the imaging FOV was constrained by simultaneous neuronal imaging in the pyramidal layer, only gliapil but no clearly detectable cell bodies of astrocytes could be identified, making those recordings less useful for comparisons of calcium signals in cell bodies versus distal processes. To show that denoising based on deep networks did not introduce artifacts, we performed a dedicated test imaging session of longer duration (33 min) and with higher laser power to increase SNR. We split the recording into two equal halves and computed reference delay maps for raw and denoised data based on the first half and performed validations on the second raw or denoised half of the recording (Supplementary Fig. 9i–l).

### Identification of propagating versus nonpropagating calcium events

To extract FOV-wide temporal delay components, the delay map was smoothed with a 2D median filter (filter size 6 µm; Fig. 6a) and then binned by rounding the delay to integer values (…, −2 s, −1 s, 0 s, +1 s, +2 s, …). Pixels across the entire FOV with the respective delays (Fig. 6b) were used to extract the mean time trace from the denoised movie (Fig. 6c). A 25-pixel-wide boundary of the FOV was discarded to prevent contamination by lateral movement artifacts. In addition, pixels that contained identified interneurons through ectopic GFAP-driven expression of GCaMP6s were excluded using manually drawn blanking masks.

The extracted temporal delay components (one time trace each for −2 s, −1 s, 0 s, +1 s, etc., pixels) were normalized as Δ*F*/*F* values (*F*_0_ defined as 20% quantile across the session) to enable a comparison between normalized traces. The average across these traces was used to identify candidate events using the findpeaks() function in MATLAB. Because an average across delay components was used as a reference to detect events, it is likely that some gliapil-only events were not identified as events. The distribution of centripetally propagating and nonpropagating events in Fig. 6d is therefore biased toward events that are also visible in processes closer to the soma. All candidate events were visually inspected and corrected. Typical events are shown in Fig. 6e. Other more complex events, which were still considered as a single event, are shown in Extended Data Fig. 7. The beginning and end of events were estimated as the trough preceding or following an event peak, again proofed by visual inspection. For each event, the mean value of each delay component was extracted, and a linear fit (*y* = *a* ⋅ *x* + *b*) was computed, with the extracted mean as *y* values and the delay in seconds as *x* values (Fig. 6c (inset)). The fit parameter *a* was normalized by *b*, the value of *y* at *x* = 0 s, resulting in the propagation slope as used throughout the Results section. This normalization has the side effect that errors due to division by small values of *b* can result in potentially erroneous large positive or negative slopes; however, at the same time, it ensures that the propagation of small-amplitude events is equally considered compared to large-amplitude events. The fit was improved by using the number of FOV pixels that contributed to each delayed trace as fit weights. To define putative astrocytic domains for single-cell analysis of delayed traces (Extended Data Fig. 8), we manually seeded cell centers based on cell bodies. This analysis could be performed only for sessions in which astrocytic cell bodies could be clearly identified and distinguished from each other and from other structures (*n* = 8 sessions). A watershed algorithm custom-written in MATLAB was used to simultaneously and iteratively expand the domains of all seed points using binary dilation until the domains encountered either the boundary of another domain or reached a distance of ≥35 µm from the seed point.

### Statistics and reproducibility

Unless otherwise indicated, nonparametric, two-sided tests were used (Mann–Whitney rank-sum test and Wilcoxon signed-rank test for unpaired and paired conditions, respectively), and no corrections for multiple testing were performed. Because results were often hierarchically organized (for example, 22 imaging sessions distributed across four animals), we computed variability within and across animals and reported the values in Supplementary Table 1. Notably, within-animal variability was similar to variability across all measurements and in general higher than across-animal variability, suggesting that the single measurements (that is, from a single imaging session) can be considered to be not strongly influenced by the batch (that is, animal identity) as quantified by intraclass correlation^78^ (Supplementary Table 1). For statistical tests on results based on hierarchically organized data, we used a recently published dedicated toolbox^79^ and applied two-sided tests unless otherwise noted. No statistical methods were used to predetermine sample sizes, but our sample sizes are similar to those reported in previous publications^3,6^. Representative results were replicated similarly with two animals (Fig. 1c,d and Extended Data Fig. 1e–g), with 22 imaging sessions from four animals (Fig. 2c), with 11 imaging sessions from four mice (Fig. 5c) and with 9 imaging sessions from four mice (Fig. 7f). Box plots used standard settings in MATLAB, with the central line at the median of the distribution, the box at the 25th and 75th percentiles and the whiskers at extreme values excluding outliers.

### Reporting summary

Further information on research design is available in the Nature Portfolio Reporting Summary linked to this article.

## Online content

Any methods, additional references, Nature Portfolio reporting summaries, source data, extended data, supplementary information, acknowledgements, peer review information; details of author contributions and competing interests; and statements of data and code availability are available at 10.1038/s41593-024-01612-8.

### Supplementary information

Supplementary Information Supplementary Figs. 1–13 and Supplementary Table 1.

Reporting Summary

Supplementary Video 1 Single-plane calcium imaging of hippocampal astrocytes in vivo*.* Calcium imaging of the calcium reporter GCaMP6s in the SO of hippocampal CA1 in a behaving mouse through an implanted cannula. The raw movie was denoised using a self-consistent denoising algorithm (DeepInterpolation; Methods). For improved display of dim astrocytic processes, some of the brighter areas in the FOV are saturated. The FOV is 600 × 600 µm^2^, and the original frame rate is 30.88 Hz (displayed at 10× speed).

Supplementary Video 2 Triple-layer calcium imaging of hippocampal astrocytes in vivo. Calcium imaging of the calcium indicator GCaMP6s in three layers of hippocampal CA1 (left: SO, middle: SP, right: SR) in a behaving mouse through an implanted cannula using a tunable lens for fast z-scanning. The raw movie was denoised using a self-consistent denoising algorithm (DeepInterpolation; Methods). The original FOV for each plane is 200 × 200 µm^2^, and the original frame rate is 10.29 Hz (displayed at 12× speed).

Supplementary Video 3 Simultaneous monitoring of astrocytic and neuronal activity, pupil changes and body movement. The extracted traces are smoothed with a five-point moving window and *z*-scored for clarity. The *x* axis indicates time in seconds. The movie is displayed at real-time speed. Related to Fig. 2.

Supplementary Video 4 Examples of mouth movement without detectable effect on astrocytes. The extracted traces are smoothed with a five-point moving window and *z*-scored for clarity. The *x* axis indicates time in seconds. The movie is displayed at 2× speed. Related to Fig. 3.

Supplementary Video 5 Examples of front limb movements (reaching and grooming) and its effect on astrocytic activity. The extracted traces are smoothed with a five-point moving window and *z*-scored for readability. The *x* axis indicates time in seconds. The movie is displayed at 2× speed. Related to Supplementary Fig. 8.

Supplementary Video 6 Self-consistent denoising of astrocytic calcium movies using DeepInterpolation. In the first part of the movie, the left version shows 140 s of a raw astrocytic calcium recording from hippocampal CA1 (SO). The right version shows the movie denoised using DeepInterpolation (Methods). In the second part of the movie, the raw calcium movie is again shown together with the denoised version, but with the raw movie smoothed using a Gaussian filter (s.d. 10 timepoints), resulting in smoothed time courses but visibly less efficient denoising compared to the self-consistent denoising method. Related to Fig. 5 and Supplementary Fig. 9.

Supplementary Video 7 Optogenetic stimulation of LC during two-photon calcium imaging in hippocampal astrocytes. Related to Fig. 7. Calcium imaging of the calcium reporter GCaMP6s in the SO of hippocampal CA1 in a behaving mouse. The yellow trace indicates the mean fluorescence across the FOV, the dot indicates the current time. The recording consists of multiple segments with 5–20 s between segments, with segment boundaries indicated by interrupted yellow traces. The raw movie was denoised using a self-consistent denoising algorithm (DeepInterpolation; Methods). The FOV is 650 × 650 µm^2^, and the original frame rate is 30.88 Hz (displayed at increased speed).

Supplementary Video 8 Stimulus-triggered average for optogenetic activation of LC during two-photon calcium imaging in hippocampal astrocytes. Stimulus-triggered averages from the data underlying Supplementary Video 7, related to Fig. 7. First part: raw fluorescence. Second part: average fluorescence, overlaid with ∆*F*/*F* in yellow. Third part: zoom-in to three selected astrocytes. Before stimulus-triggered averaging, the raw movie was denoised using a self-consistent denoising algorithm (DeepInterpolation; Methods). The FOV is 650 x 650 µm^2^, and the original frame rate is 30.88 Hz (displayed at increased speed).

### Source data

Source data for Fig. 1 Raw data for Fig. 1f,g,i,j,l.

Source data Fig. 2 Raw data for single-cell data and for behavioral data.

Source data Fig. 3 Raw data for Fig. 3c,d,f–h,j,k.

Source data Fig. 4 Raw data for Fig. 4a–g.

Source data Fig. 5 Raw data for Fig. 5a,b,I,k,m.

Source data Fig. 6 Raw data for Fig. 6c,d,f.

Source data Fig. 7 Raw data for Fig. 7b,e,g,j–l,n–q.

Source data Fig. 8 Raw data for Fig. 8b–d,f–h,j,k.

Source data Extended Data Fig. 1 Raw data for Extended Data Fig. 1c,d.

Source data Extended Data Fig. 2 Raw data for Extended Data Fig. 2a–c.

Source data Extended Data Fig. 3 Raw data for Extended Data Fig. 3a–f.

Source data Extended Data Fig. 7 Raw data for Extended Data Fig. 7a–f.

Source data Extended Data Fig. 8 Raw data for Extended Data Fig. 8d–f.

Source data Extended Data Fig. 9 Raw data for all time traces for the four cells.

Source data Extended Data Fig. 10 Raw data for all observables and neuronal recordings.

## Data Availability

Example raw data of astrocytic calcium imaging together with MATLAB and Python programs to compute delay maps as shown in Fig. 5 have been made available on GitHub (https://github.com/HelmchenLabSoftware/Centripetal_propagation_astrocytes) and at a Zenodo repository^41^. A subset of the raw data for a single imaging session is available at a Zenodo repository due to space limitations^80^. Full datasets are available from the corresponding authors upon request. Source data are provided with this paper.

## Code availability

Demo programs to compute delay maps from raw astrocytic calcium imaging data, as shown in Fig. 5, are provided via GitHub together with example data under https://github.com/HelmchenLabSoftware/Centripetal_propagation_astrocytes and at a Zenodo repository^41^. Example code is provided in both MATLAB and Python. All other custom codes used for analyses described in the manuscript will be available from the corresponding authors upon request.
