# Supplementary material for: Centripetal integration of past events in hippocampal astrocytes regulated by locus coeruleus
Source: Nat Neurosci. 2024 Apr 3;27(5):927–39. doi: 10.1038/s41593-024-01612-8 (PMC11089000; doi:10.1038/s41593-024-01612-8)
Supplement: Supplementary file 1 — Supplementary Figs. 1–13 and Supplementary Table 1. [file 41593_2024_1612_MOESM1_ESM.pdf]

nature neuroscience

Article

https://doi.org/10.1038/s41593-024-01612-8

Centripetal integration of past events in hippocampal astrocytes regulated by locus coeruleus

In the format provided by the
authors and unedited

Supplementary information

Centripetal integration of past events in hippocampal astrocytes regulated by locus coeruleus

by

Peter Rupprecht, Sian N Duss, Denise Becker, Christopher M Lewis,

Johannes Bohacek, Fritjof Helmchen

Table of contents

- Supplementary Figure S1. Manual selection of astrocytic active ROIs (related to Fig. 1).
- Supplementary Figure S2. Unmixing of astrocytic signals from neuronal calcium recordings.
- Supplementary Figure S3. Global astrocytic activity can be well explained by past behavior, mean neuronal spike rate or pupil diameter.
- Supplementary Figure S4. Predictions of global astrocytic activity based on combined and higher-dimensional regressors.
- Supplementary Figure S5. Performance for different regressors across sessions is highly correlated.
- Supplementary Figure S6. Instantaneous and time-dependent co-variation of behavioral, neuronal and astrocytic signals.
- Supplementary Figure S7. Regression of astrocytic activity using past locomotion and movement.
- Supplementary Figure S8. Global astrocytic calcium increases during non-locomotor movements (reaching).
- Supplementary Figure S9. Pixel-wise computation of delays is improved by self-supervised de-noising. Illustration of the workflow (panels a-h). Validation of delay maps based on raw vs. denoised data (panels i-l).
- Supplementary Figure S10. Centripetal propagation replicated using diverse methods.
- Supplementary Figure S11. Early and late astrocytic components and neuronal activity, related to Fig. 6c.
- Supplementary Figure S12. Slope of astrocytic global events as a function of calcium event magnitude and of a 'saliency score'.
- Supplementary Figure S13. Correlation functions between global astrocytic activity and paw movement, extension of Fig. 8h.
- Supplementary Table 1. Within- and across-animal variance for hierarchically acquired data.
- Movies 1-8

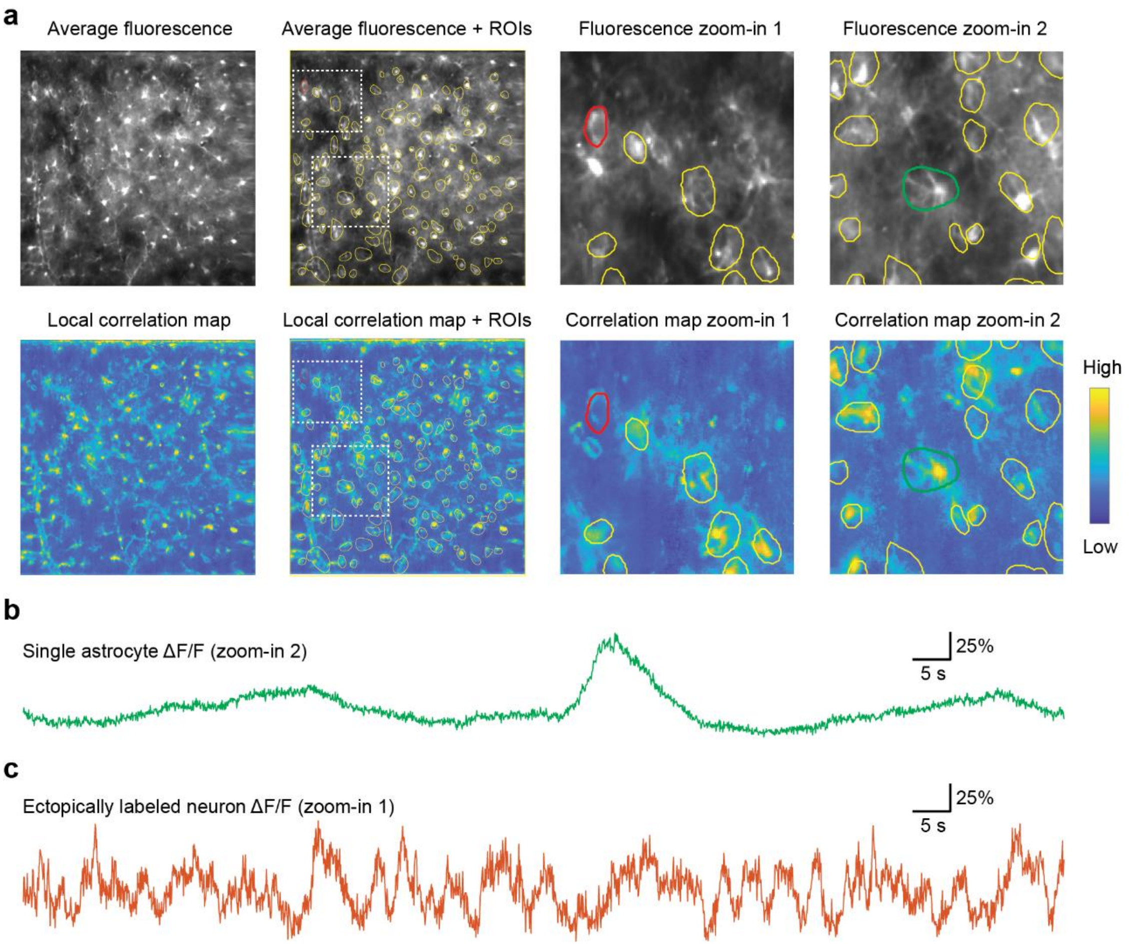

Figure S1 | Manual selection of astrocytic active ROIs (related to Fig. 1). a, ROIs were selected based on mean fluorescence (structural label, top row) and a map of local correlations (functional label, bottom row). High values in the map of local correlations indicate structural components that are activated in a coherent manner. During manual selection of ROIs, temporal components for manually selected ROIs were displayed (see panels b and c), allowing, *e.g.*, to quickly recognize and discard ROIs of ectopically labeled interneurons. **b,** Example of a temporal component extracted from an astrocytic ROI centered around a soma (zoom-in 2 from (a), green ROI). **c,** Example of a temporal component extracted from an interneuron ROI (zoom-in 1 from (a), red ROI), displaying the fast-varying nature of neuronal as opposed to astrocytic signals.

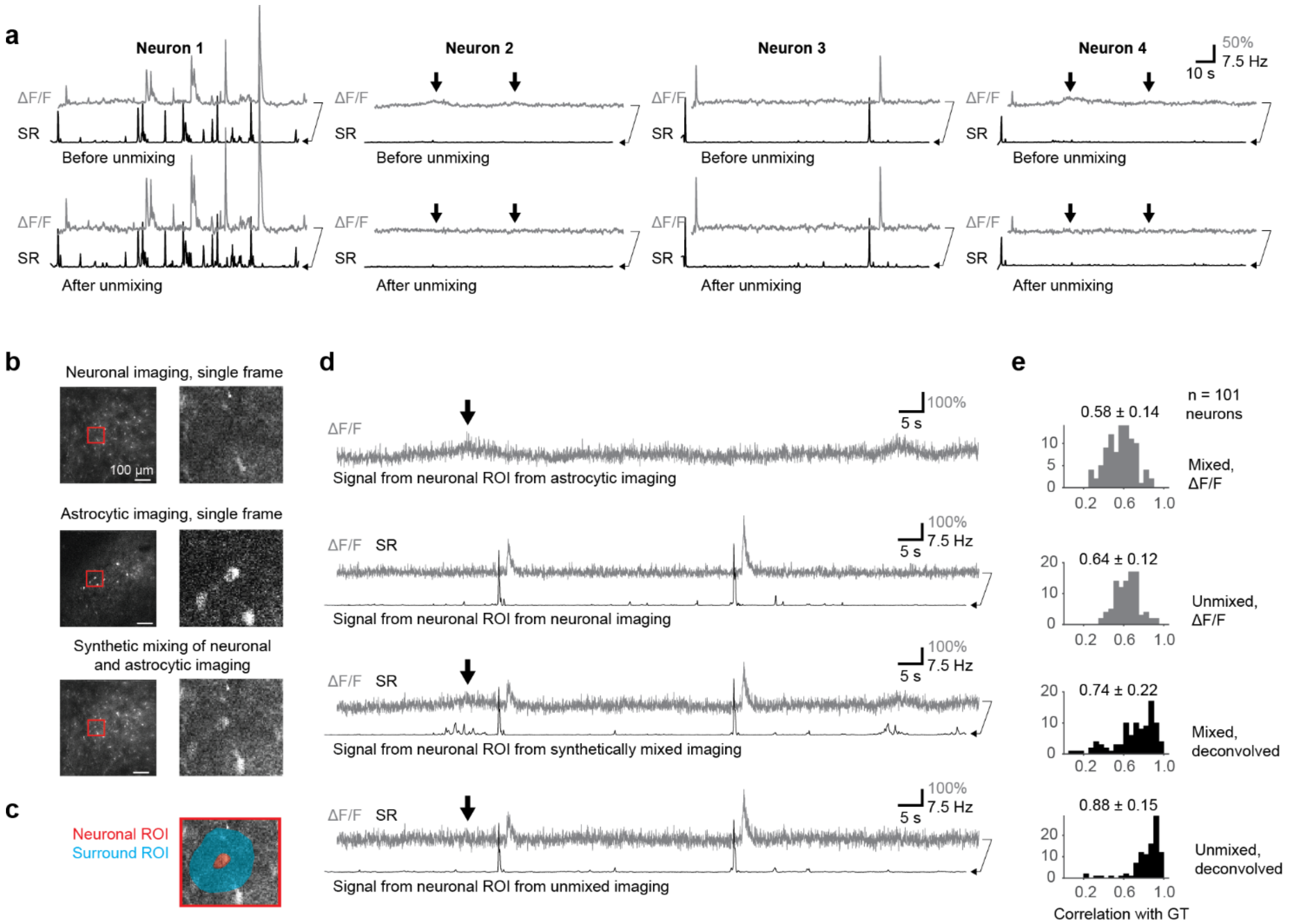

Figure S2 | Unmixing of astrocytic signals from neuronal calcium recordings. As astrocytes and neurons were recorded simultaneously using indicators with overlapping non-distinguishable spectra (GCaMP6s and GCaMP6f), we took advantage of the fact that the two cell types were spatially separated (astrocytes in the *stratum oriens*, neurons in the pyramidal layer) and that typical time courses of mean activity patterns were clearly distinct. Astrocytic signals were not visibly contaminated by neuronal signals, as evidenced by the lack of a 0 s-peak in the correlation function (Fig. 4a). Neuronal signals were, however, contaminated by surrounding gliapil. We performed unmixing of astrocytic signals using linear regression of the surrounding gliapil (Methods). To visualize and validate our approach, we show here examples of unmixed neuronal imaging data **(panel a)** and synthetically mixed and afterwards unmixed signals **(panels b-e)**. **a,** Typical examples for neuronal traces (ΔF/F and deconvolved spike rate SR) before and after unmixing. For many neurons, unmixing has no clearly visible effect (neurons 1 and 3) since there is only sparsely distributed gliapil in the pyramidal cell layer. For some neurons, contamination can be observed as a slow transient that is clearly distinguishable from calcium transients that originate from neuronal spikes (neuron 2 and 4). In this case, deconvolution, which is trained to detect fast onset transients, removes the slow transients (neuron 2). Slow transients are also removed by unmixing and further suppressed by deconvolution (neurons 2 and 4). Therefore, both unmixing and deconvolution remove astrocytic contamination when present. Deconvolved traces are shifted to the left for visualization purposes. **b,** Single frames of separate neuronal imaging data (top), astrocytic imaging data (middle) and the synthetically mixed data (bottom). **c,** Illustration of neuronal soma ROI (red) and surrounding contamination ROI (blue). **d,** Time traces derived – from top to bottom – from astrocytic imaging, from neuronal imaging, from synthetically mixed imaging data and from the unmixed imaging data, with the associated deconvolved traces in black (if applicable). Deconvolved traces are shifted to the left for visualization purposes. **e,** Distributions of correlation values for ΔF/F traces before and after unmixing (correlation to the ΔF/F traces of the original neuronal imaging data before synthetic mixing) and of correlation values for deconvolved traces obtained from data before and after unmixing (correlation to the original deconvolved traces). Each data point of this distribution corresponds to a neuron and the correlation of its extracted time trace with the reference extracted from the original neuronal imaging data.

Together, both unmixing and deconvolution contribute to recover ground truth. All differences between the four distributions (mixed vs. unmixed ΔF/F; mixed vs. unmixed deconvolved; mixed ΔF/F vs. mixed deconvolved; unmixed ΔF/F vs. unmixed deconvolved) in panels (e) were statistically significant (paired Wilcoxon test, $p<10^{-10}$ after Bonferroni correction for multiple comparisons).

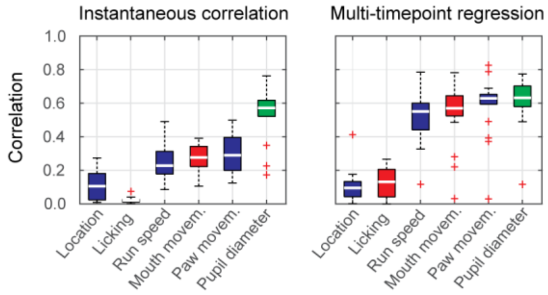

Figure S3 | Global astrocytic activity can be well explained by past behavior, mean neuronal spike rate or pupil diameter. Same analysis as in Fig. 3c,d but for a different set of animals, in which calcium imaging was performed in astrocytes only (thus lacking the predictions by neuronal ・F/F and SR; 19 imaging sessions from 3 animals). Results are similar to the results for the 4 animals shown in Fig. 3c,d.

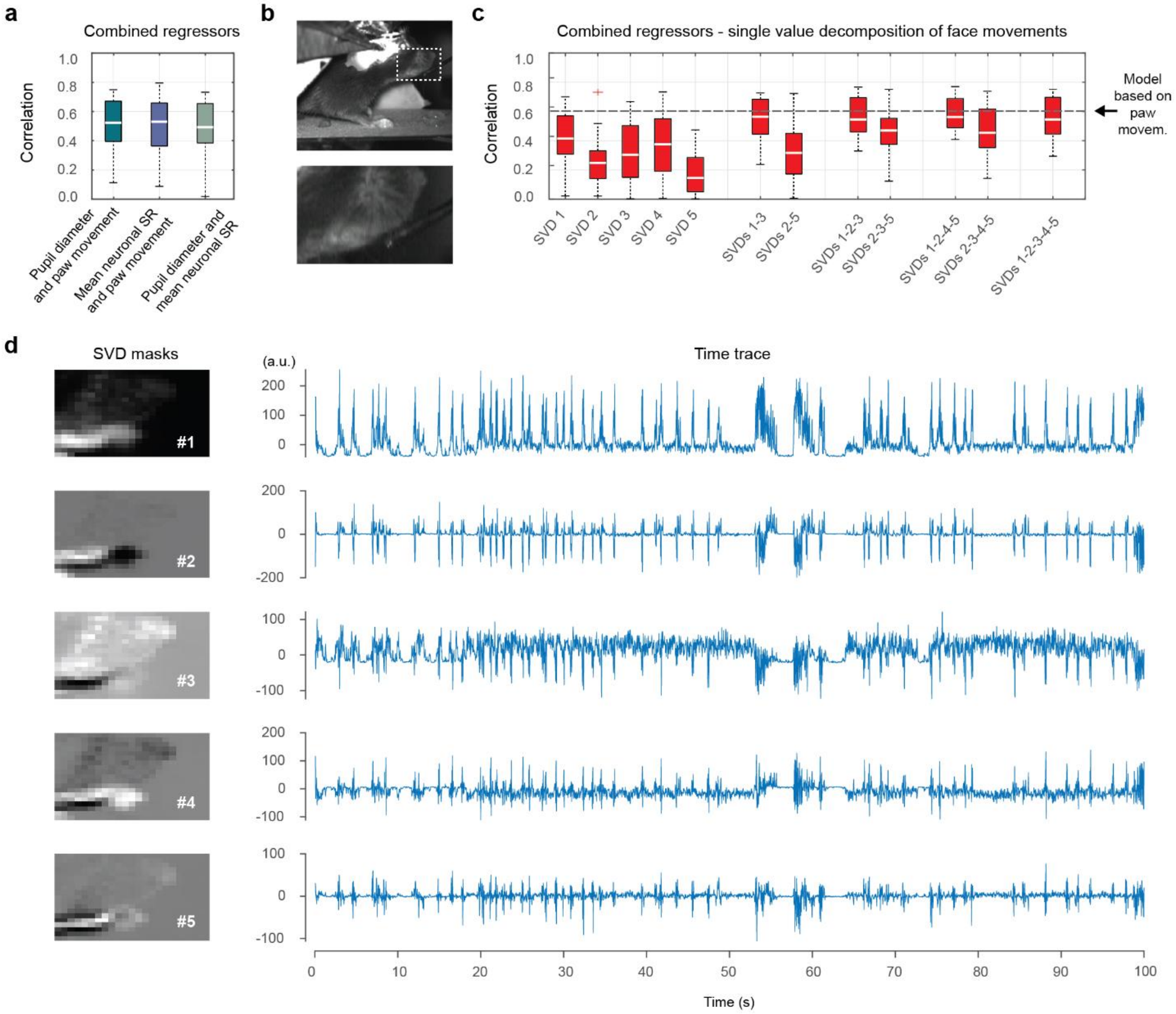

Figure S4 | Predictions of global astrocytic activity based on combined and higher-dimensional regressors. a, Using combined regressors (*e.g.,* pupil diameter and paw movement) instead of a single regressor (*e.g.,* only pupil diameter) did not improve predictions, as measured by correlation values (y-axis). **b,** Using the face recording and its movement components (SVD) to explain global astrocytic activity. **c,** Predictions when combining single or multiple regressors (SVDs) extracted from face movement using Facemap[15]. Example spatial and temporal components are shown in panel (d). No combination of multiple face movement components resulted in predictions better than from simple paw movement regression (single- instead of multi-dimensional regressor). All results measured across 22 sessions for 4 animals. **~~c~~d**, Visualization of spatial and temporal components for mouth movement analysis for an excerpt of a single imaging session. The lack of improvement when using multiple or higher-dimensional regressors (panels (a) and (c)) is likely due to two effects. First, the auto-correlated astrocytic signal yields relatively few independent data points that result in overfitting when increasing the number of regressors and deterioration of cross-validated predictions. Second, distinct regressors lead to highly similar predictions due to redundant signals (Fig. 3e,f), resulting in lack of strong improvements when combining regressors.

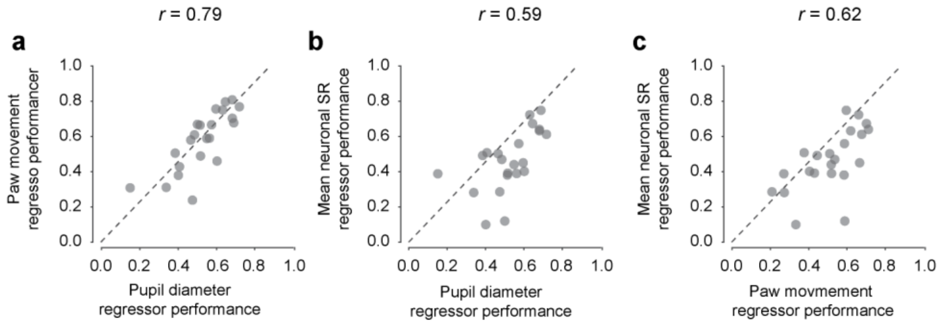

Figure S5 | Performance for different regressors across sessions is highly correlated. Extension of Fig. 3g. **a,** Performance of pupil diameter vs. paw movement as regressors. **b,** Performance of pupil diameter vs. mean neuronal spike rate (SR) as regressors. **c,** Performance of paw movement vs. mean neuronal spike rate as regressors. For all panels, a single data point represents the average performance of the cross-validated dilated linear regression averaged for an imaging session. Values (*r*) indicate the correlation between the two regressors' performances. The dashed lines represent the identity relationship.

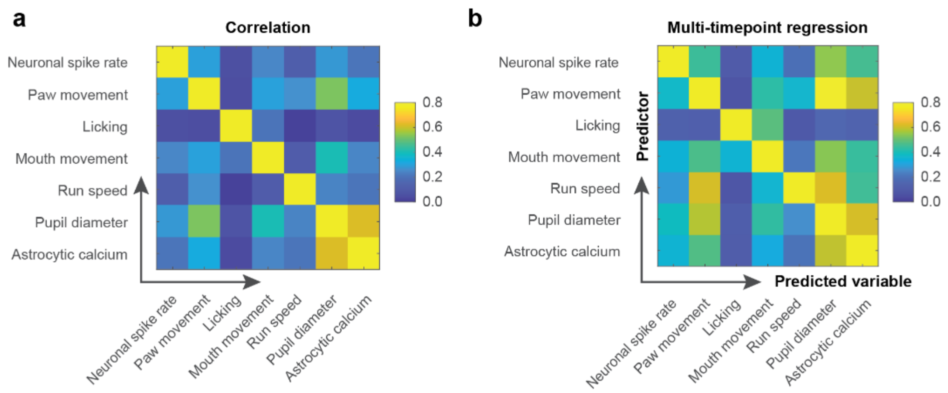

Figure S6 | Instantaneous and time-dependent co-variation of behavioral, neuronal and astrocytic signals. Extension of Fig. 3c-d. **a,** Correlation of each observed variable with each of the other observed variables. Most pairs of variables (with few exceptions: pupil diameter vs. astrocytic calcium and paw movement vs. pupil diameter) are only weakly correlated. The right-most column corresponds to the median values in Fig. 3a. **b,** Multi-timepoint regression as described in Fig. 3 was used to predict a variable (column) from each of the other variables (row). The right-most column corresponds to the median values in Fig. 3d. By the definition of correlation and correlation functions (concepts that are directly related to variance explained), slowly-varying signals (*e.g.*, astrocytic mean activity or pupil diameter) are easier to predict than quickly varying signals (*e.g.*, mean neuronal activity). It is therefore natural, and should be considered for any interpretation, that a quickly varying signal like mean neuronal activity explains a large fraction of the variance of astrocytic calcium but not vice versa. All values were computed as medians across 22 sessions from 4 animals.

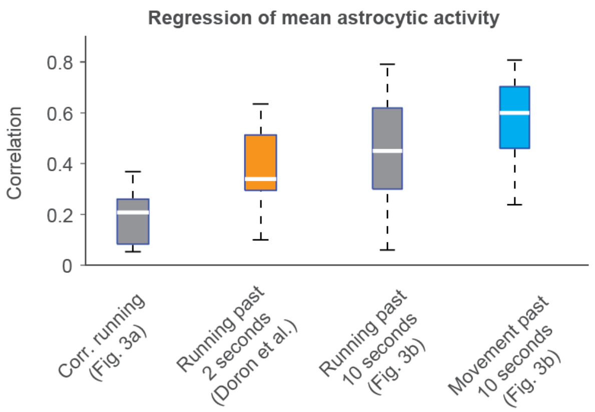

Figure S7 | Regression of astrocytic activity using past locomotion and movement. From left to right. (1, gray) Instantaneous correlations explains only a small amount of variance of astrocytic activity. (2, orange) Using the past 2 seconds of running explains a higher but still low amount of variance of astrocytic activity; this quantification uses the time window for the past running speed that was used in a previous study (ref. 6), and lead to the wrong conclusion that past running cannot explain astrocytic activity well. (3, gray) Using the past 10 seconds of running explains on average a higher amount of variance compared to using the 2-s time window. (4, blue) Using the past 10 seconds of body movement, not restricted to locomotion, further increased the variance explained dramatically. In conclusion, using running and body movement from more temporally distant time points (>2 s, <10 s) increased the variance explained of mean astrocytic activity.

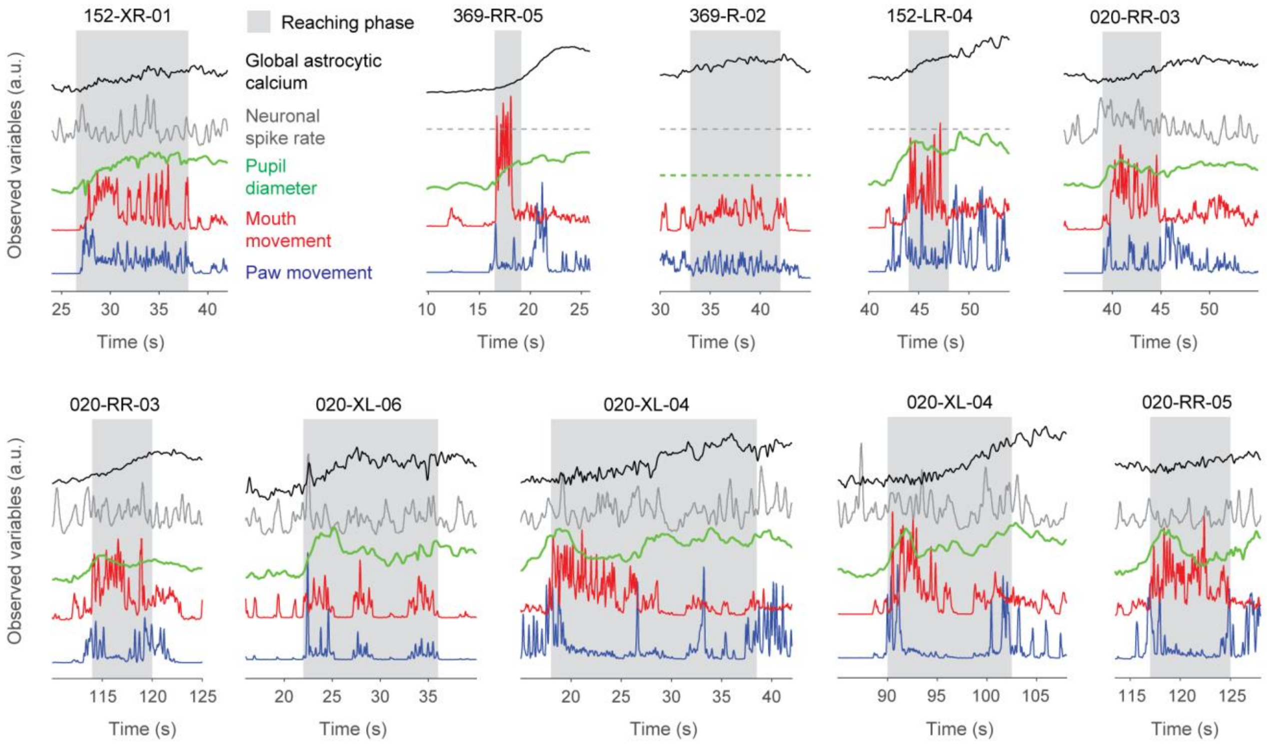

Figure S8 | Global astrocytic calcium increases during non-locomotor movements (reaching). Simultaneously recorded variables are color-coded and represented with dashed flat lines if no recordings were available. Panel titles indicate mouse ID (*e.g.,* 152-XR for the first panel) and the respective imaging session (*e.g.,* 01). The (manually defined) grasping phase is highlighted as gray background. Reaching, which resulted in paw movement close to the lick spout in proximity of the mouth, is usually visible as deflections of the "mouth movement" (red). All events in this figure are shown, together with the recorded variables and the behavioral video in Movie 5.

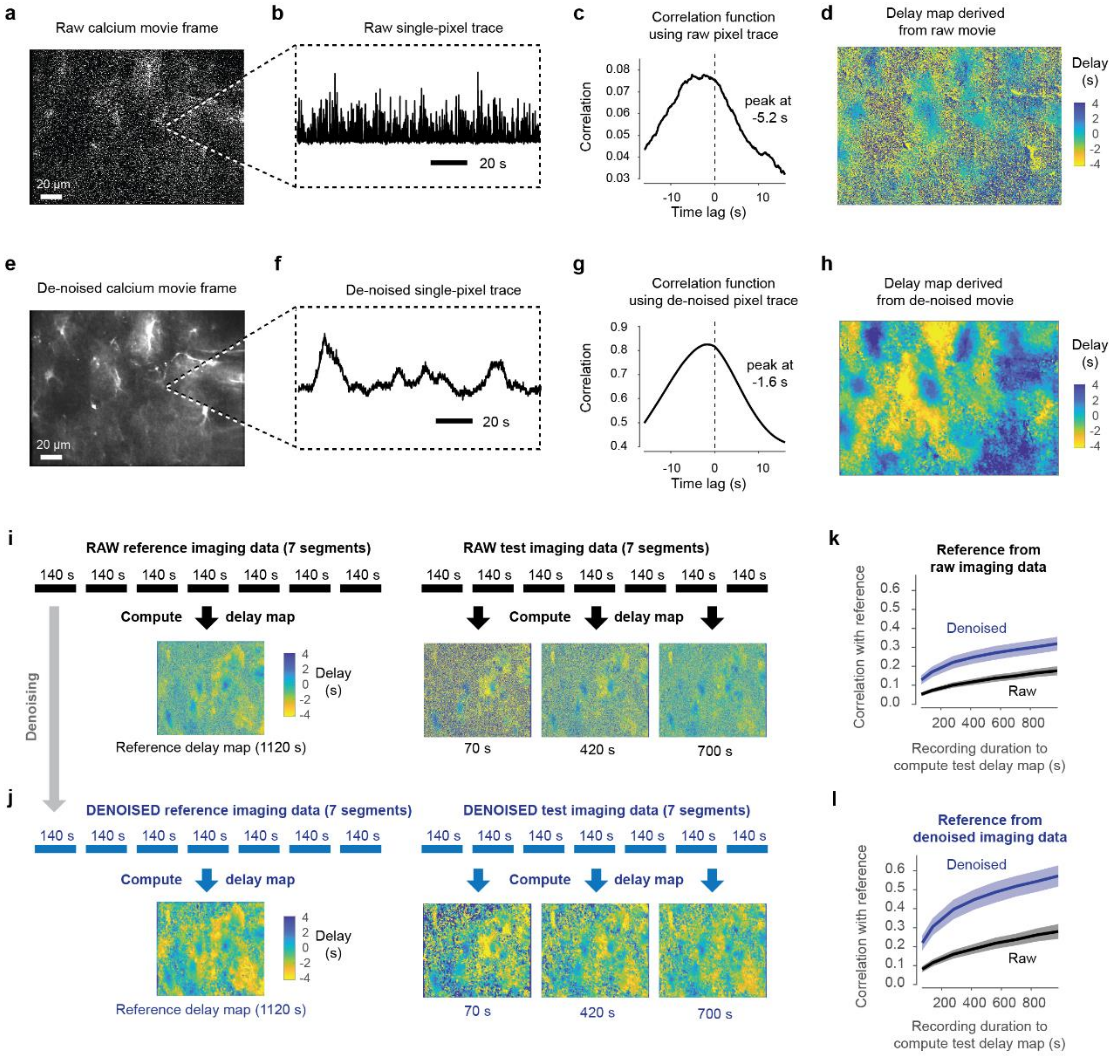

Figure S9 | Pixel-wise computation of delays is improved by self-supervised de-noising. Illustration of the workflow (panels a-h). The top row (a-d) shows the results of the processing pipeline as described in Fig. 5e but without de-noising of the raw movie. The bottom row (e-h) shows the results using the de-noised movie **a,** Single frame of the raw movie. **b,** Single-pixel trace extracted from the raw movie in (a). **c,** Correlation function computed from the pixel trace in (b) with the global astrocytic activity across the entire FOV. The peak of the correlation function is, in this case, detected at -5.2 s. **d,** Delays for all pixel traces, mapped onto the imaging FOV. **e-h,** Same as (a-d) but based on the de-noised movie. The resulting delay map (h) is less noisy than in (d). **Validation of delay maps based on raw vs. denoised data (panels i-l).** A recording from the same FOV comprising 14 segments of each 140 s duration was acquired and split into two equal halves. The first half was used to compute a reference delay map. A second delay map (test delay map) was computed based on a varying number of segments (0.5 - 7 segments) and then compared to the reference map. **i,** Reference map and example test delay maps computed from the raw recording are shown. **j,** Reference map and example test delay maps computed from the denoised recording are shown. **k,** Similarity of test delay maps to the reference map computed from the *raw* recordings. The similarity increases with the number of segments used to compute the test delay map but is always higher when computed from denoised recordings. Shaded corridors indicate the standard deviation across 7 imaging FOVs. **l,** Similarity of test delay maps to the reference map computed from the *denoised* recordings. Shaded corridors indicate the standard deviation across 7 imaging FOVs. Again, the similarity increases with the number of segments used to compute

the test delay map but is always higher when computed from denoised recordings. These analyses show that delay maps converge (1) when more frames are used for their computation and (2) when the underlying recordings are denoised.

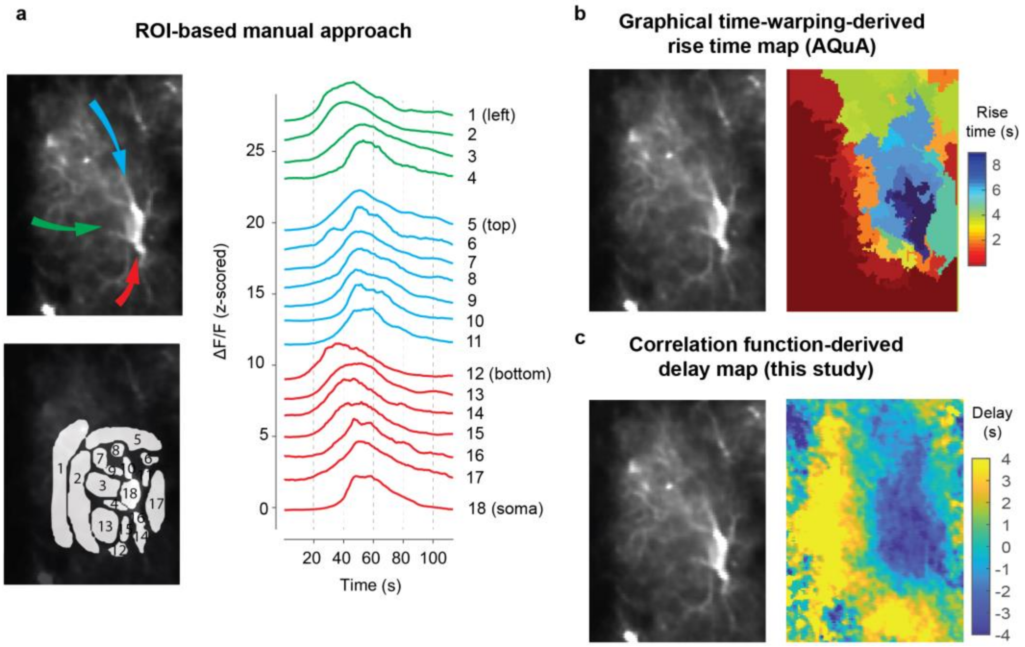

Figure S10 | Centripetal propagation replicated using diverse methods. This event in this specific astrocyte was chosen for a comparative analysis since the spatio-temporal dynamics were clearly distinct by eye. Only in this case, the alternative methods (ROI-based approach, AQuA) yielded useable results. The event is therefore not representative but showcases that under conditions of excellent SNR and clearly discernible spatio-temporal events, our approach based on correlation function-derived delay maps is consistent with other analysis approaches. **a,** ROI-based approach. ROIs were manually drawn with an interactive ROI-tool (ref. 69) based on spatially distinct time traces. The ROIs were manually grouped to reveal three distinct pathways of calcium signal propagation towards the soma (along the blue, green and red arrows). Earliest activation is seen from the red processes (ROIs 12-17) and the green processes (ROIs 1-4), while the blue processes (ROIs 5-11) are activated only slightly before the soma. **b,** Rise time map derived using the graphical time-warping method implemented in AQuA (ref. 21). Briefly, parameters in AQuA were adjusted to label only one single event that comprises the entire astrocyte and its processes. To make this work with AQuA, which is not designed to detect such global events, the denoised movie was fed into the toolbox in temporally reversed order. From the event, toolbox parameters were adjusted until sub-events were reasonably large in their spatial extent and not noisy. Then, the rise time map based on graphical time warping was extracted. NaN values in the resulting rise time map were filled by valid values of nearest neighboring pixels. **c,** Delay map based on the maximum of pixel-wise correlation functions computed between the respective pixel trace and the global mean activity, as described in Fig. 5.

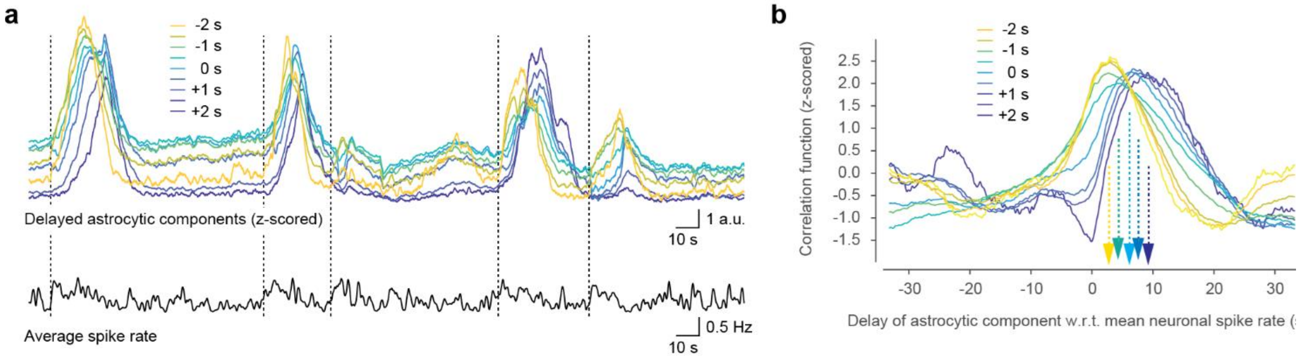

Figure S11 | Early and late astrocytic components and neuronal activity, related to Fig. 6c. a, Example of delayed components extracted from a FOV as defined in Fig. 6 (colored traces) together with the simultaneously recorded average deconvolved spike rate (black, below). Prior to onset of visually detectable astrocytic events (dashed vertical lines), neuronal spike rates tend to be transiently increased. Also the earliest astrocytic component (yellow) is increased only after this transient increase of neuronal activity. Astrocytic traces were z-scored for better visualization of dynamics. **b,** Separate correlation functions of early and late astrocytic components with simultaneously recorded mean spike rates (average across 12 sessions from 4 mice; averaging was performed in a weighted manner according to the number of pixels associated with a respective delayed component). The correlation functions between 2.5 s (earliest component) and 9.0 s (latest astrocytic component) after neuronal activity. Due to the long auto-correlation time of astrocytic and also neuronal activity (Fig. 4), only the peak of the correlation functions can be meaningfully interpreted.

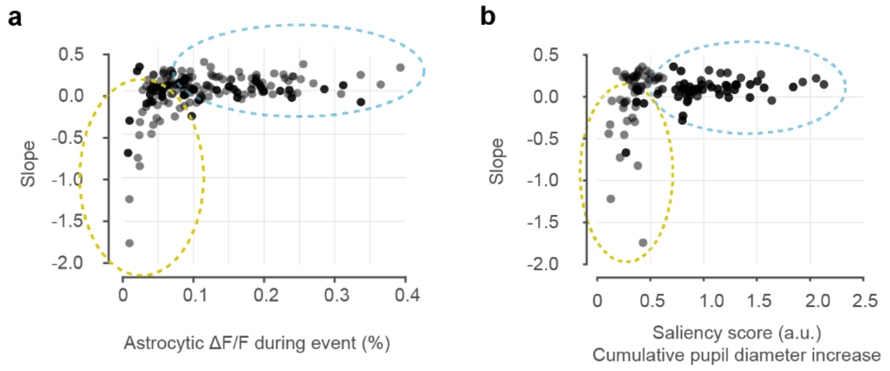

Figure S12 | Slope of astrocytic global events as a function of calcium event magnitude and of a 'saliency score'. Panels are analogous to panels Fig. 6d, but use the astrocytic mean ΔF/F value (a) or the saliency score (b) instead of pupil diameter as the dependent variable (x-axis). The saliency score is computed from the sum of the rectified derivative of the measured pupil diameter in the time window during the event, but shifted to the past by 2 seconds. The score therefore measures the amount of (positive) pupil change, instead of pupil diameter itself.

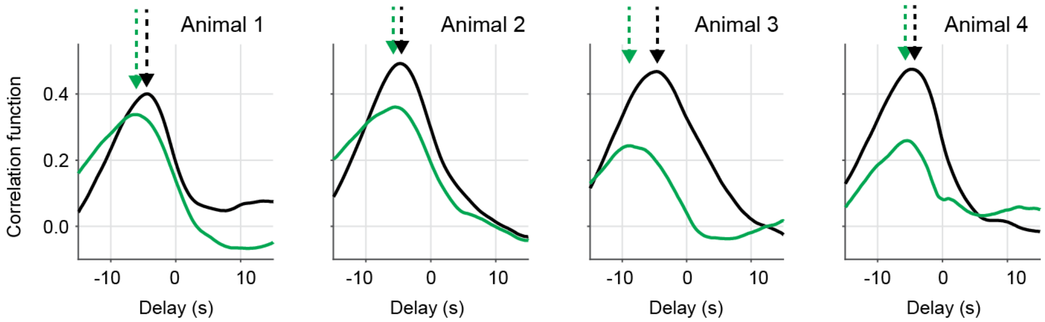

Figure S13 | Correlation functions between global astrocytic activity and paw movement, extension of Fig. 8h. The peak of the correlation function indicates how much later global astrocytic activity occurred after paw movement. A longer delay indicates that the signaling pathways for global astrocytic signals and centripetal propagation were slower. Delays computed from correlation functions with prazosin injected-animals (green) were longer than delays from control animals (black) for all 4 animals. Control recordings were performed with injection of a vehicle, prazosin injections with injection of the same amount of solution with prazosin dissolved on the subsequent day. Each correlation function was computed from a recording session of 7 segment of 140 s each (980 s total). Extracted delays computed from all paired correlation functions (maximum value) are plotted in the box plot in Fig. 8h.

	Variable	Median across all data points	S.D. across all pairs	S.D. across animals	S.D. within animals	ICC
**Figure 1i**: Pairwise correlation	Correlation	0.72	0.20	0.09	0.21	0.155172
**Figure 1l**: Pairwise correlation	*oriens-oriens*	0.5912	0.1642	0.0583	0.1339	0.159362
	pyramidale-pyramidale	0.6099	0.1947	0.0793	0.0789	0.502528
	radiatum-radiatum	0.6583	0.1333	0.0499	0.1564	0.09239
	oriens-pyramidale	0.5731	0.1823	0.0172	0.0878	0.036958
	oriens-radiatum	0.5381	0.1640	0.1519	0.0972	0.709489
	pyramidale-radiatum	0.5735	0.1896	0.0020	0.1102	0.000329
**Figure 3c**: Correlation	Pupil	0.6147	0.1214	0.1223	0.1133	0.538145
	Paws	0.3227	0.1255	0.0917	0.1268	0.34340
	Run	0.2080	0.1069	0.0857	0.0851	0.503513
	SR	0.2075	0.0967	0.0774	0.0770	0.502591
	Mouth	0.2459	0.0962	0.0660	0.0750	0.436429
	ΔF/F	0.1693	0.1299	0.1259	0.0847	0.688420
	Licking	0.0355	0.0580	0.0373	0.0469	0.387449
	Location	0.0136	0.1114	0.0206	0.0587	0.109652
**Figure 3d**: Multi-timepoint regression	Pupil	0.6069	0.1544	0.0974	0.1557	0.281262
	Paws	0.5997	0.1687	0.0764	0.1711	0.166238
	Running	0.4596	0.1734	0.1070	0.1666	0.292032
	SR	0.4498	0.2011	0.0858	0.2017	0.153225
	Mouth	0.4415	0.1741	0.1435	0.1504	0.476536
	ΔF/F	0.2915	0.1856	0.1379	0.1693	0.398843
	Licking	0.1381	0.2195	0.1416	0.1661	0.420879
	Location	0.2067	0.1296	0.0717	0.0656	0.544341
**Figure 3h**: Future and past	Pupil past	0.5983	0.1507	0.1052	0.1505	0.32823
	Paws past	0.6051	0.1729	0.0876	0.1670	0.215781
	SR past	0.4682	0.1789	0.1068	0.1684	0.286843
	Pupil future	0.5669	0.1670	0.1353	0.1638	0.405571
	Paws future	0.3243	0.1922	0.1517	0.1893	0.391061
	SR future	0.1739	0.1777	0.1451	0.1455	0.498624
**Figure 3j**: DGL	Pupil DGL	0.5809	0.1416	0.1249	0.1203	0.518754
	Paws DGL	0.6480	0.1488	0.0999	0.1422	0.330455
	SR DGL	0.5360	0.1627	0.0856	0.1517	0.241506
**Figure 4**: Delays	Neurons spike rate	4.2167	3.3913	1.22240	2.8253	0.157679
	Paw movement	3.6667	1.2760	0.76277	0.94236	0.395832
	Licking	3.6667	3.2866	0.99943	1.2708	0.382150
	Mouth movement	3.6333	1.5115	1.66980	1.3221	0.614665
	Run speed	3.3667	1.5704	1.25090	1.4083	0.441016
	Pupil diameter	1.5000	1.0573	0.78489	0.74105	0.528706
**Figure 8**: Spike rates	Awake (Hz)	0.14081	0.031215	0.020572	0.026125	0.382743
	Anesthetized (Hz)	0.0930	0.0110	0.0110	-	0

Supplementary Table 1 | Within- and across-animal variance for hierarchically acquired data. Details are described in the Statistics subsection of the Methods section. Intra-class-correlation (ICC) is defined as the across-animal variance divided by across-animal variance plus within-animal variance. "S.D." indicates the standard deviation as a measure for variability.

Movie captions

Movie 1 | Single-plane calcium imaging of hippocampal astrocytes *in vivo.* Calcium imaging of the calcium reporter GCaMP6s in the *stratum oriens* of hippocampal CA1 in a behaving mouse through an implanted cannula. The raw movie was denoised using a self-consistent denoising algorithm (DeepInterpolation, see Methods). For improved display of dim astrocytic processes, some of the brighter areas in the field of view are saturated. The field of view is 600 x 600 μm^2, the original frame rate 30.88 Hz (displayed at 10x speed).

Movie 2 | Triple-layer calcium imaging of hippocampal astrocytes *in vivo*. Calcium imaging of the calcium indicator GcaMP6s in three layers of hippocampal CA1 (left: *stratum oriens*, middle: *stratum pyramidale*, right: *stratum radiatum)* in a behaving mouse through an implanted cannula using a tunable lens for fast z-scanning. The raw movie was denoised using a self-consistent denoising algorithm (DeepInterpolation, see Methods). The original field of view for each plane is 200 x 200 μm^2, the original frame rate 10.29 Hz (displayed at 12x speed).

Movie 3 | Simultaneous monitoring of astrocytic and neuronal activity, pupil changes and body movement. The extracted traces are smoothed with a 5-point moving window and z-scored for clarity. The x-axis indicates time in seconds. The movie is displayed at real-time speed. Related to Figure 2.

Movie 4 | Examples of mouth movement without detectable effect on astrocytes. The extracted traces are smoothed with a 5-point moving window and z-scored for clarity. The x-axis indicates time in seconds. The movie is displayed at 2x speed. Related to Figure 3.

Movie 5 | Examples of front limb movements (reaching and grooming) and its effect on astrocytic activity. The extracted traces are smoothed with a 5-point moving window and z-scored for readability. The x-axis indicates time in seconds. The movie is displayed at 2x speed. Related to Supplementary Fig. S8.

Movie 6 | Self-consistent denoising of astrocytic calcium movies using DeepInterpolation. In the first part of the movie, the left version shows 140 s of a raw astrocytic calcium recording from hippocampal CA1 (*stratum oriens*). The right version shows the movie denoised using DeepInterpolation (see Methods). In the second part of the movie, the raw calcium movie is again shown together with the denoised version, but with the raw movie smoothed using a Gaussian filter (standard deviation 10 time points), resulting in smoothed time courses but visibly less efficient denoising compared to the self-consistent denoising method. Related to Figures 5 and Supplementary Fig. S9.

Movie 7 | Optogenetic stimulation of locus coeruleus during two-photon calcium imaging in hippocampal astrocytes. Related to Figure 7. Calcium imaging of the calcium reporter GCaMP6s in the *stratum oriens* of hippocampal CA1 in a behaving mouse. The yellow trace indicates the mean fluorescence across the FOV, the dot indicates the current time. The recording consists of multiple segments with 5-20 s between segments, with segment boundaries indicated by interrupted yellow traces. The raw movie was denoised using a self-consistent denoising algorithm (DeepInterpolation,

see Methods). The field of view is 650 x 650 μm^2, the original frame rate 30.88 Hz (displayed at increased speed).

Movie 8 | Stimulus-triggered average for optogenetic activation of locus coeruleus during two-photon calcium imaging in hippocampal astrocytes. Stimulus-triggered averages from the data underlying Movie 7, related to Figure 7. First part: raw fluorescence. Second part: Average fluorescence, overlaid with $\Delta F/F$ in yellow. Third part: Zoom-in to three selected astrocytes. Before stimulus-triggered averaging, the raw movie was denoised using a self-consistent denoising algorithm (DeepInterpolation, see Methods). The field of view is 650 x 650 μm^2, the original frame rate 30.88 Hz (displayed at increased speed).
