# Supplementary material for: Centripetal integration of past events in hippocampal astrocytes regulated by locus coeruleus
Source: Nat Neurosci. 2024 Apr 3;27(5):927–39. doi: 10.1038/s41593-024-01612-8 (PMC11089000; doi:10.1038/s41593-024-01612-8)
Supplement: Supplementary file 2 — Reporting Summary [file 41593_2024_1612_MOESM2_ESM.pdf]

nature portfolio

Corresponding author(s): Peter Rupprecht, Fritjof Helmchen

Last updated by author(s): Jan 22, 2024

Reporting Summary

Nature Portfolio wishes to improve the reproducibility of the work that we publish. This form provides structure for consistency and transparency in reporting. For further information on Nature Portfolio policies, see our Editorial Policies and the Editorial Policy Checklist.

Statistics

For all statistical analyses, confirm that the following items are present in the figure legend, table legend, main text, or Methods section.

n/a	Confirmed	
☐	☒	The exact sample size (*n*) for each experimental group/condition, given as a discrete number and unit of measurement
☐	☒	A statement on whether measurements were taken from distinct samples or whether the same sample was measured repeatedly
☐	☒	The statistical test(s) used AND whether they are one- or two-sided<br>*Only common tests should be described solely by name; describe more complex techniques in the Methods section.*
☐	☒	A description of all covariates tested
☐	☒	A description of any assumptions or corrections, such as tests of normality and adjustment for multiple comparisons
☐	☒	A full description of the statistical parameters including central tendency (e.g. means) or other basic estimates (e.g. regression coefficient) AND variation (e.g. standard deviation) or associated estimates of uncertainty (e.g. confidence intervals)
☐	☒	For null hypothesis testing, the test statistic (e.g. *F*, *t*, *r*) with confidence intervals, effect sizes, degrees of freedom and *P* value noted<br>*Give P values as exact values whenever suitable.*
☒	☐	For Bayesian analysis, information on the choice of priors and Markov chain Monte Carlo settings
☐	☒	For hierarchical and complex designs, identification of the appropriate level for tests and full reporting of outcomes
☐	☒	Estimates of effect sizes (e.g. Cohen's *d*, Pearson's *r*), indicating how they were calculated

Our web collection on statistics for biologists contains articles on many of the points above.

Software and code

Policy information about availability of computer code

Data collection	All calcium imaging data were collected using custom-written software written in C++ (http://rkscope.sourceforge.net/, Chen et al., eLife, 2021). Behavioral videos were captured using IC-Capture 2.5 (The Imaging Source). Additional behavioral variables (running, location on the treadmill, timing of rewards and air puffs) were recorded using custom-written code in Python 3 as described in the Methods section.
Data analysis	For ROI-based analysis of neuronal and astrocytic calcium imaging data, a custom-written script was used to manually extract ROIs (https://github.com/PTRRupprecht/Drawing-ROIs-without-GUI, Rupprecht et al., Neuron, 2018). Further custom-written scripts were used to unmix neuronal and astrocytic components, as described in the Methods section. These analysis steps were performed in Matlab 2020b.<br>Extracted raw calcium traces were converted to estimated spike rates using an algorithm based on supervised deep learning, implemented in Python 3 (Rupprecht et al., Nature Neuroscience, 2021).<br>Modeling of astrocytic activity as a function of other variables using linear regression or using a simulated differential equation (Fig. 3) was costum-implemented in Matlab 2020b.<br>To denoise astrocytic calcium imaging data (Fig. 5 and 6), we used an algorithm based on self-supervised deep learning, implemented in Python 3 (Lecoq et al., Nature Methods, 2021).<br>For unbiased analysis of spatiotemporal astrocytic dynamics ("delay maps"; Fig. 5 & 6), we computed correlation functions with the global activity trace for each pixel of the denoised movie using an algorithm custom-written in Matlab, described in the Methods section and provided together with demo data on Github: https://github.com/HelmchenLabSoftware/Centripetal_propagation_astrocytes. This software is archived in its current version permanently at Zenodo: https://doi.org/10.5281/zenodo.10420895.

For manuscripts utilizing custom algorithms or software that are central to the research but not yet described in published literature, software must be made available to editors and reviewers. We strongly encourage code deposition in a community repository (e.g. GitHub). See the Nature Portfolio guidelines for submitting code & software for further information.

Data

Policy information about availability of data

All manuscripts must include a data availability statement. This statement should provide the following information, where applicable:

- Accession codes, unique identifiers, or web links for publicly available datasets
- A description of any restrictions on data availability
- For clinical datasets or third party data, please ensure that the statement adheres to our policy

Example raw data of astrocytic calcium recordings are available on Github upon publication of the manuscript under https://github.com/HelmchenLabSoftware/Centripetal_propagation_astrocytes. This data example together with its Github repository is archived in its current version permanently at Zenodo: https://doi.org/10.5281/zenodo.10420895. A subset of the raw data for a single imaging session is available at a Zenodo repository due to space limitations: https://doi.org/10.5281/zenodo.10558021. All other data used in the manuscript are available from the corresponding authors upon request.

Human research participants

Policy information about studies involving human research participants and Sex and Gender in Research.

Reporting on sex and gender	No human subjects participated in this study.
Population characteristics	No human subjects participated in this study.
Recruitment	No human subjects participated in this study.
Ethics oversight	No human subjects participated in this study.

Note that full information on the approval of the study protocol must also be provided in the manuscript.

Field-specific reporting

Please select the one below that is the best fit for your research. If you are not sure, read the appropriate sections before making your selection.

☒ Life sciences ☐ Behavioural & social sciences ☐ Ecological, evolutionary & environmental sciences

For a reference copy of the document with all sections, see nature.com/documents/nr-reporting-summary-flat.pdf

Life sciences study design

All studies must disclose on these points even when the disclosure is negative.

Sample size	Sample sizes were not predetermined for this study. In total, 41 experimental sessions across 6 animals were recorded for the descriptive experiments covered in Figs. 1-6. This number of sessions was necessary to capture the experimentally observed behavioral variance (cf. the relatively broad distributions in Fig. 3). Since the results were similarly variable across imaging sessions within animals as compared to imaging sessions across animals (cf. Suppl. Table 1), we analyzed several imaging sessions per animal (see also the "Statistics" subsection in the "Methods" section). For optogenetic experiments, 4 animals were used and results replicated across animals (Fig. 7d-o; Fig. 8). For prazosin i.p. experiments, results were examined across 4 animals. For experiments with low variance of the main outcome (e.g., anesthesia experiments, cf. Fig. 8a-d, Extended Data Fig. 10), a smaller sample number of sessions was recorded (4 sessions across 2 animals).
Data exclusions	Data from mice with unstable hippocampal windows (visible axial movement of the brain during body movement) were not analyzed. Data from these mice were excluded from all analyses.<br>For the analysis of spatio-temporal dynamics in single astrocytic domains (Extended Data Fig. 8), sessions where the astrocytic somata in the FOV could not be clearly defined and visually distinguished from other structures were not included in the analysis, as described in the Methods section. For the analysis in Supplementary Fig. S11, only sessions were included with clearly identifiable astrocytic somata to provide the full range of astrocytic delay components.
Replication	The main findings of our study (global and local events in astrocytes, Fig. 1f,h,i,j,l; variance of global astrocytic activity explained by other variables, Fig. 3; temporal sequence of events, Fig. 4; centripetal propagation, Fig. 5; conditional centripetal propagation, Fig. 6; optogenetic stimulation of centripetal propagation, Fig. 7; anesthesia- and prazosin induced reduction of astrocytic responses, Fig. 8) were replicated across sessions and across mice. Behavioral variability was discussed explicitly in the manuscript (e.g., Fig. 3g or Fig. 8f). Outlier observations not well covered by the main analyses were discussed in the main text (text related to the arrows in Extended Data Fig. 4) or shown as supplementary figure (Extended Data Fig. 7). Our main finding (centripetal propagation) was replicated using different imaging modalities (3D vs. 2D, Extended Data Fig. 6), using different analysis approaches (AQuA or ROI-based vs. our delay map approach, Supplementary Fig. S10) and with different pre-processing (raw data vs. denoised, Supplementary Fig. S9).
Randomization	Animals were not allocated into different groups in this study.
Blinding	Anaimals were not allocated into different groups and no blinding was therefore necessary. The experimenter was not blinded to data analysis

Blinding	since this would have been impractical due to complex algorithms that were developed during ongoing analyses and tailored towards the questions of interest.

Reporting for specific materials, systems and methods

We require information from authors about some types of materials, experimental systems and methods used in many studies. Here, indicate whether each material, system or method listed is relevant to your study. If you are not sure if a list item applies to your research, read the appropriate section before selecting a response.

Materials & experimental systems

n/a	Involved in the study	
☐	☒	Antibodies
☒	☐	Eukaryotic cell lines
☒	☐	Palaeontology and archaeology
☐	☒	Animals and other organisms
☒	☐	Clinical data
☒	☐	Dual use research of concern

Methods

n/a	Involved in the study	
☒	☐	ChIP-seq
☒	☐	Flow cytometry
☒	☐	MRI-based neuroimaging

Antibodies

Antibodies used	Primary AB rabbit-anti-GFAP, DAKO, Cat# Z0334, RRID: AB_10013382<br>Secondary AB anti-rabbit with Cy3, Jackson ImmunoResearch Labs, Cat# 711-165-152, RRID:AB_2307443
Validation	The primary anti-GFAP antibody was used before to visualize GFAP expression in multiple publications, including ones focused on the hippocampus, e.g., Zhang et al., eLife (2019) https://doi.org/10.7554/eLife.45303

Animals and other research organisms

Policy information about studies involving animals; ARRIVE guidelines recommended for reporting animal research, and Sex and Gender in Research

Laboratory animals	4-6 month-old C57BL/6J mice and genetically modified mice (Thy1-GCaMP6f, GP5.17, Dana et al., PloS One, 2014; C57BL/6-Tg(Dbh-iCre)1Gsc, Parlato et al., Development, 2007) of both sexes were used.
Wild animals	No wild animals were used in this study.
Reporting on sex	Experiments were conducted in animals of both sexes (total of 8 male mice, 6 female mice). The main results were observed in both sexes, but sex was not a variable included in study design. Due to the relatively low number of animal per sex, sex-dependent strengths of the observed effects were not investigated.
Field-collected samples	No samples were collected from the field for this study.
Ethics oversight	All experimental procedures were carried out in accordance with the guidelines of the Federal Veterinary Office of Switzerland and were approved by the Cantonal Veterinary Office in Zurich.

Note that full information on the approval of the study protocol must also be provided in the manuscript.
